# Proteoglycan inhibition of canonical BMP-dependent cartilage maturation delays endochondral ossification

**DOI:** 10.1242/dev.201716

**Published:** 2024-01-12

**Authors:** Elham Koosha, Connor T. A. Brenna, Amir M. Ashique, Niteesh Jain, Katie Ovens, Toshiyasu Koike, Hiroshi Kitagawa, B. Frank Eames

**Affiliations:** ^1^ Department of Anatomy, Physiology, and Pharmacology, University of Saskatchewan, Saskatoon, SK S7N 5E5, Canada; ^2^ Department of Computer Science, University of Calgary, Calgary, AB T2N 1N4, Canada; ^3^ Department of Biochemistry, Kobe Pharmaceutical University, Higashinada-ku, Kobe 658-0003, Japan

**Keywords:** Proteoglycans, BMP signalling, Cartilage maturation, Endochondral ossification, Developmental timing, Zebrafish

## Abstract

During endochondral ossification, chondrocytes secrete a proteoglycan (PG)-rich extracellular matrix that can inhibit the process of cartilage maturation, including expression of *Ihh* and *Col10a1*. Because bone morphogenetic proteins (BMPs) can promote cartilage maturation, we hypothesized that cartilage PGs normally inhibit BMP signalling. Accordingly, BMP signalling was evaluated in chondrocytes of wild-type and PG mutant (*fam20b^−/−^*) zebrafish and inhibited with temporal control using the drug DMH1 or an inducible dominant-negative BMP receptor transgene (*dnBMPR*). Compared with wild type, phospho-Smad1/5/9, but not phospho-p38, was increased in *fam20b^−/−^* chondrocytes, but only after they secreted PGs. Phospho-Smad1/5/9 was decreased in DMH1-treated or *dnBMPR*-activated wild-type chondrocytes, and DMH1 also decreased phospho-p38 levels. *ihha* and *col10a1a* were decreased in DMH1-treated or *dnBMPR*-activated chondrocytes, and less perichondral bone formed. Finally, early *ihha* and *col10a1a* expression and early perichondral bone formation of *fam20b* mutants were rescued with DMH1 treatment or *dnBMPR* activation. Therefore, PG inhibition of canonical BMP-dependent cartilage maturation delays endochondral ossification, and these results offer hope for the development of growth factor therapies for skeletal defects of PG diseases.

## Teaser

**Summary:** Proteoglycan inhibition of BMP signalling in zebrafish cartilage regulates the timing of a common bone-forming process.

## INTRODUCTION

During endochondral ossification, cartilage and bone development are intimately linked. Cartilage forms and then undergoes a tightly regulated process called maturation (Eames et al., 2003). Mature chondrocytes express collagen type X (ColX) and Indian hedgehog (Ihh), the latter of which induces bone formation in the adjacent perichondrium (Vortkamp et al., 1996; St-Jacques et al., 1999; Hammond and Schulte-Merker, 2009). Reflecting the functional link between cartilage and bone during endochondral ossification, changes to the timing of perichondral bone formation can be caused by changes to the timing of cartilage maturation (Hammond and Schulte-Merker, 2009; Eames et al., 2011; St-Jacques et al., 1999; Bi et al., 2001; Mis et al., 2014). Molecules coordinating these events continue to be discovered.

Growth factors in the bone morphogenetic protein (BMP) family have been associated with skeletal development since their identification over 50 years ago, having roles in both chondrocyte and osteoblast differentiation (Hogan, 1996; Wozney and Rosen, 1998). During endochondral ossification, BMPs and their receptors are expressed in chondrocytes and the perichondrium (Yazaki et al., 1998). BMP signalling induces chondrocyte differentiation from mesenchymal cells, and at later stages also promotes cartilage maturation, inducing cellular hypertrophy and *Ihh* expression (Volk et al., 2000; Seki and Hata, 2004; Yi et al., 2000; Steinert et al., 2009; Zhang et al., 2003; Shum et al., 2004). In addition, BMPs support cell commitment to the osteoblast lineage and further progression of osteoblast differentiation (Lian et al., 2006; Yamaguchi et al., 2000).

BMPs act through a complex of two kinds of BMP receptor, known as types I and II, to modulate at least two intracellular signalling pathways (Katagiri and Watabe, 2016). Upon ligand binding to the receptor complex, type I BMP receptors (BMPRIs), commonly known as ALKs, or activin receptor-like kinases, are phosphorylated by type II receptors (Rosenzweig et al., 1995). So far, four BMPRIs have been identified: ALK1 (ACVRL1), ALK2 (ACVR1), ALK3 (BMPRIA) and ALK6 (BMPRIB) (Luo, 2017). Whereas ALK3 and ALK6 are relatively specific to BMPs, ALK2 also signals upon binding with activins, other members of the TGFβ superfamily (Gomez–Puerto et al., 2019). BMPRIs determine the specificity of downstream signalling along one of two intracellular signalling pathways: the canonical Smad pathway (specifically Smad1/5/9) and the noncanonical p38 MAP kinase pathway (Derynck and Zhang, 2003). Upon phosphorylation by activated BMP receptors, Smad1/5/9 or p38 (Mapk14) move from the cytoplasm to the nucleus, where they act as transcription factors to regulate gene expression (Derynck and Zhang, 2003). Although *in vitro* experiments are abundant (e.g. Saitta et al., 2019; Shang et al., 2016; Van Caam et al., 2015), more limited *in vivo* studies suggest that cartilage maturation uses canonical BMP signalling. For example, cartilage-specific loss of Smad1 and Smad5 has been shown to inhibit collagen type X expression and chondrocyte hypertrophy (Retting et al., 2009).

**Fig. 1.**
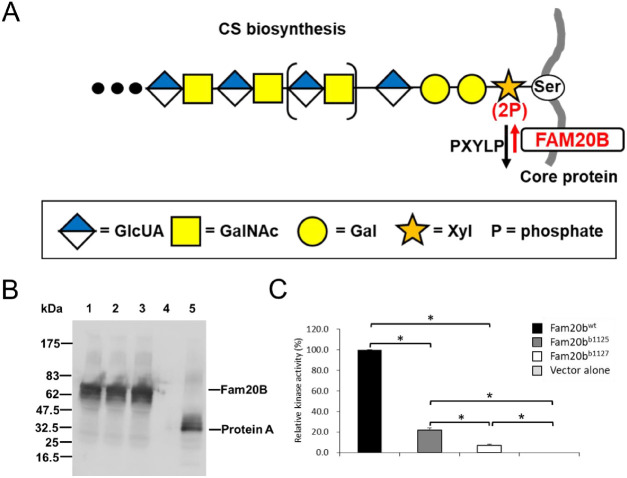
**Mutant Fam20b proteins have severely hypomorphic kinase activities.** (A) Model illustrating that Fam20b transiently phosphorylates xylose, the first sugar added to a serine residue of the core protein during synthesis of a chondroitin sulfate PG (Koike et al., 2009). 2-Phosphoxylose phosphatase (PXYLP) removes the phosphate, thus promoting efficient glycosaminoglycan side chain outgrowth (Koike et al., 2014). (B) Culture medium from COS-1 cells transfected with secreted forms of zebrafish Fam20b or vector alone was incubated with IgG-Sepharose, and proteins purified from the medium were subjected to SDS-PAGE. Expression of each protein A-tagged protein was examined with anti-mouse IgG antibody. Lane 1: Fam20b^wt^; lane 2: Fam20b^b1125^; lane 3: Fam20b^b1127^; lane 4: protein marker; lane 5: vector alone. (C) After normalization from immunoblots in B, evaluation of kinase activity demonstrated that the mutant kinases Fam20b^b1125^ and Fam20b^b1127^ both have a significant decrease in activity compared with Fam20b^wt^, with Fam20b^b1127^ displaying significantly less activity than Fam20b^b1125^. Both mutant kinases, however, showed more activity than the negative, empty-vector control. Data are mean+s.e.m. (*n*=3, Tukey's multiple comparison test, *P<0.05). CS, chondroitin sulfate; Gal, galactose; GalNAc, N-acetylgalactosamine; GlcUA, glucuronic acid; P, phosphate; Ser, serine; Xyl, xylose.

Extracellularly, BMP signalling can be altered through BMP interaction with proteoglycans (PGs) in the extracellular matrix (ECM) (Brown and Eames, 2016). PGs are composed of core proteins to which large glycosaminoglycan side chains are added (Fig. 1A). The specific impact of PGs on BMP signalling can be positive or negative, because PGs can either facilitate interaction of BMPs and their receptors, acting as co-receptors, or restrict the bioavailability of BMPs through such mechanisms as limiting their diffusion (Jiao et al., 2007; Matsumoto et al., 2010). Perhaps owing to the abundance of PGs expressed by cartilage, PG mutations can affect BMP signalling during endochondral ossification. As an example of negative regulation of BMP signalling by PGs, a mouse model of multiple hereditary exostoses with a mutation in the PG synthesis gene *Ext1* has osteochondromas that are associated with increased BMP signalling in the perichondrium (Inubushi et al., 2017). Also, syndecan 3 and other PGs normally reduce the amount of BMP accessible for signalling during early stages of chondrogenesis (Fisher et al., 2006). As an example of positive regulation of BMP signalling by PGs, polydactyly and other skeletal abnormalities in Simpson–Golabi–Behmel dysmorphia syndrome are caused by mutations in the PG glypican 3 that limit a cellular response to BMP4 (Paine-Saunders et al., 2000).

The timing of endochondral ossification is altered in many PG mutants, likely through changes to cartilage maturation. For example, Ext loss-of-function models in mice and zebrafish show delayed endochondral ossification linked to delays in cartilage maturation (Clement et al., 2008; Hilton et al., 2005; Koziel et al., 2004). Mutation in UDP-xylose synthase 1, which regulates PG sugar precursor production, also delays cartilage maturation and endochondral ossification (Eames et al., 2010). A mutation in bisphosphate nucleotidase 2 (previously called *Jaws*) disrupts PG sulfation and causes delayed cartilage maturation (Frederick et al., 2008; Sohaskey et al., 2008). In contrast to these delays in endochondral ossification, other PG mutations accelerate the process. Mutation of aggrecan, the most abundant PG in cartilage, causes early chondrocyte hypertrophy and *COL10A1* expression in chick (Domowicz et al., 2009). Also, mutation in the PG synthesis gene xylosyltransferase 1 in mouse or zebrafish accelerates cartilage maturation and perichondral bone formation (Mis et al., 2014; Eames et al., 2011).

How PGs affect BMP-dependent cartilage maturation is unclear, as published data show both positive and negative regulation. Loss of either *Ext1* or the PG synthesis gene *Chsy1* increases BMP/Smad signalling, chondrocyte hypertrophy, and *Col10a1* and *Mmp13* expression (Kawashima et al., 2020; Lyu et al., 2022). However, BMP signalling might be decreased during cartilage maturation when the PG sulfation gene carbohydrate sulfotransferase 11 (previously called chondroitin-4-sulfotransferase 1) is mutated, as decreased phospho-Smad1 staining was reported (Klüppel et al., 2005).

*fam20b* mutant zebrafish offer an opportunity to clarify the relationship between PGs, BMP signalling and cartilage maturation. Fam20b is a kinase that phosphorylates the initiator xylose residue of the linkage sugar tetrasaccharide of PGs, which is a rate-limiting step in PG synthesis (Koike et al., 2009; Wen et al., 2014). *fam20b* is expressed specifically in cartilage, and mutant *fam20b* cartilage has lower levels of PGs than that of wild-type siblings (Eames et al., 2011). *fam20b* mutants produce early perichondral bone because they initiate cartilage maturation earlier. For example, *ihha* and *ihhb* transcripts are expressed earlier in chondrocytes of the *fam20b* mutants, and crossing an *ihha* mutation into *fam20b* mutants blocked the formation of early perichondral bone (Eames et al., 2011).

Given that *fam20b* mutants have early cartilage maturation, which BMPs can promote (Volk et al., 2000; Steinert et al., 2009; Zhang et al., 2003; Shum et al., 2004; Seki and Hata, 2004), we hypothesized that cartilage PGs normally delay endochondral ossification by inhibiting BMP-dependent cartilage maturation. To test this hypothesis, canonical and non-canonical BMP signalling were evaluated in chondrocytes of wild-type and *fam20b^b1127^* zebrafish using immunofluorescence. Also, BMP signalling was inhibited with temporal control to focus on cartilage maturation using either the drug DMH1 or a heat shock-inducible dominant-negative BMP receptor transgenic line [*Tg(hsp70l:dnXla.Bmpr1a-GFP)w30*; Pyati et al., 2005; Hao et al., 2010]. *fam20b* mutant chondrocytes had increased canonical BMP signalling, and, for the first time, skeletal defects in a PG mutant were rescued by blocking BMP signalling. These experiments demonstrate clearly that cartilage PGs normally delay the timing of endochondral ossification by inhibiting canonical BMP-dependent cartilage maturation. We also discuss the relevance of these findings to osteoarthritis, during which loss of cartilage PGs and ectopic cartilage maturation occurs (Pitsillides and Beier, 2011).

## RESULTS

### Zebrafish *fam20b* mutants encode severe biochemical hypomorphs

Fam20b is a xylose kinase that promotes the addition of glycosaminoglycan side chains to the core protein of PGs, such as the chondroitin sulfate PGs that are abundant in cartilage ECM (Fig. 1A) (Koike et al., 2009; Wen et al., 2014). Even though mutations in zebrafish *fam20b* decrease, but do not abrogate, PG production *in vivo* (Eames et al., 2011), the biochemical functions of mutant forms of Fam20b have not been assessed directly *in vitro*. To test kinase activity, *fam20b^wt^*, *fam20b^b1125^* and *fam20b^b1127^* were each cloned into an expression vector that caused the kinases to be secreted after transfection into COS-1 cells. Following purification from culture medium and normalization for protein amounts (Fig. 1B), kinase activity was determined with an artificial substrate, Galb1-4Xylβ1-O-ITI, *in vitro* (Koike et al., 2009, 2022). Compared with Fam20b^wt^*,* the mutant kinases Fam20b^b1125^ and Fam20b^b1127^ both demonstrated a significant reduction in activity (Fig. 1C). Fam20b^b1127^ had significantly lower activity than Fam20b^b1125^, with only 6.8% of the activity of Fam20b^wt^, although both mutant kinases had significantly more than zero, which was the relative activity of the negative, empty-vector control (Fig. 1C). Therefore, zebrafish with the severe hypomorph *fam20b^b1127^* were used for the remainder of experiments.

### Canonical Smad-mediated BMP signalling increases as *fam20b^−/−^* chondrocytes secrete PGs

To evaluate whether BMP signalling was active in developing cartilage of zebrafish, laser capture microdissection of cranial cartilage was performed, avoiding perichondral cells, followed by RNA sequencing (Gomez-Picos et al., 2022; Nguyen et al., 2023). Several members of the BMP signalling pathway were expressed in cranial cartilage of 6 days post-fertilization (dpf) wild-type zebrafish larvae (Table S1). For example, BMP receptors (*bmpr1aa*, *bmpr1ab*, *bmpr2a* and *bmpr2b*), BMP ligands (*bmp2b*, *bmp6* and *bmp8a*) and BMP intracellular mediators (*smad1*, *smad5*, *smad6b*, *id1*, *id2a*, *smurf1* and *smurf2*) were all expressed above threshold levels. These data support the idea that BMP signalling can be autocrine within cartilage, since both BMP ligands and receptors are expressed by chondrocytes.

**Fig. 2.**
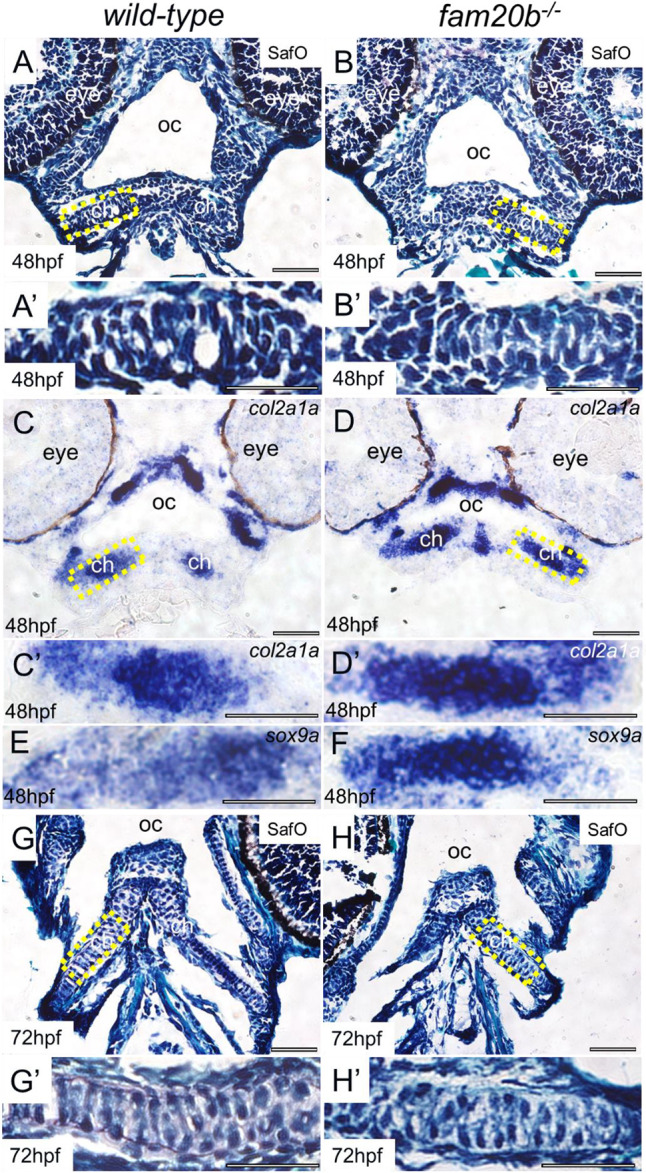
**Histological and molecular markers show similar timing of early cartilage formation between wild types and *fam20b* mutants.** (A-B′) Lack of Safranin O staining demonstrated that developing ceratohyal cells of wild-type and *fam20b* mutant zebrafish did not secrete cartilage PGs at 48 hpf. (C-F) Cells in the mesenchymal condensation of both wild types (C,C′,E) and *fam20b* mutants (D,D′,F) were specified to form cartilage, as evidenced by *col2a1a* (C-D′) and *sox9a* (E,F) expression. (G-H′) The spacing between chondrocytes suggested that cartilage PG secretion had occurred in ceratohyal chondrocytes of both wild-type and *fam20b* mutant zebrafish at 72 hpf, but light Safranin O staining was only observed in wild types. Scale bars: 50 μm. ch, ceratohyal; oc, oral cavity; SafO, Safranin O.

Early stages of cartilage formation appeared similar in wild types and *fam20b* mutants. Safranin O staining on cryosections demonstrated that developing ceratohyal cells had not secreted many PGs at 48 h post-fertilization (hpf) in either wild-type or *fam20b* mutant zebrafish embryos (Fig. 2A-B′). Cells in mesenchymal condensations of the ceratohyal for both wild types and *fam20b* mutants expressed the early cartilage markers *col2a1a* and *sox9a* (Fig. 2C-F). Previous work demonstrated that both wild-type and *fam20b* mutant chondrocytes secrete cartilage PGs by 72 hpf, but at decreased levels (Eames et al., 2011). Similarly, the spacing between developing ceratohyal chondrocytes had increased in both wild types and *fam20b* mutants by 72 hpf, although faint Safranin O staining was only visible in wild types (Fig. 2G-H′).

**Fig. 3.**
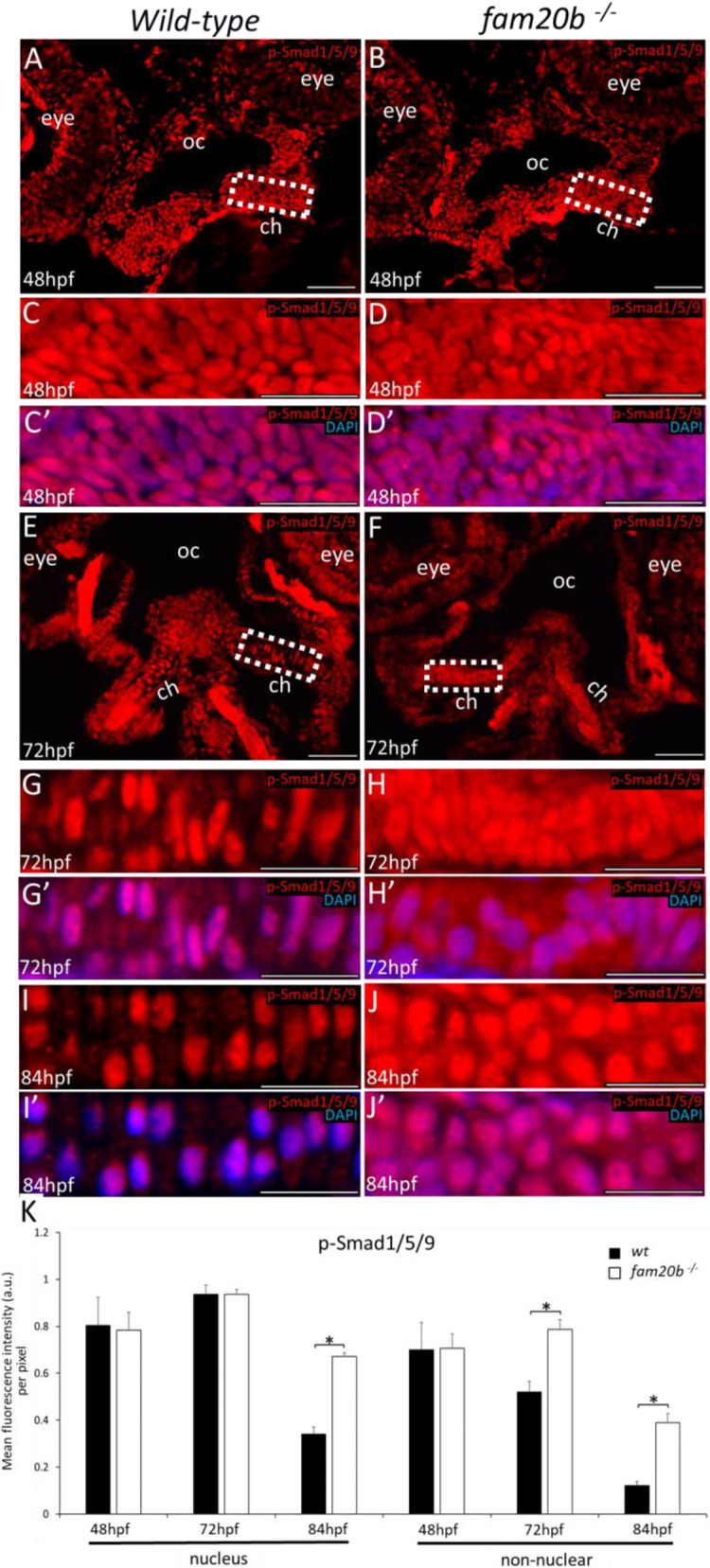
**Smad-mediated BMP signalling is increased in *fam20b^−/−^* chondrocytes.** (A-D′) Fluorescence immunostaining showed no clear differences between wild types (A,C,C′) and *fam20b* mutants (B,D,D′) in the levels of p-Smad1/5/9 in nuclei and non-nuclear regions of condensing ceratohyal mesenchyme at 48 hpf. (E-H′) p-Smad1/5/9 immunoreactivity appeared higher in non-nuclear regions of *fam20b**^−/−^*** ceratohyal chondrocytes (F,H,H′) at 72 hpf, compared with wild types (E,G,G′). (I-J′) p-Smad1/5/9 immunoreactivity appeared increased in both the nuclei and non-nuclear regions of *fam20b**^−/−^*** chondrocytes (J,J′), compared with wild types (I,I′), at 84 hpf. (K) Quantitative image analyses (*n*=3 for each group) demonstrated significant increases in p-Smad1/5/9 levels of *fam20b**^−/−^*** chondrocytes (*P<0.05, one-way ANOVA and paired Student's *t*-test). Scale bars: 50 μm. a.u., arbitrary units; ch, ceratohyal; oc, oral cavity; wt, wild type.

**Fig. 4.**
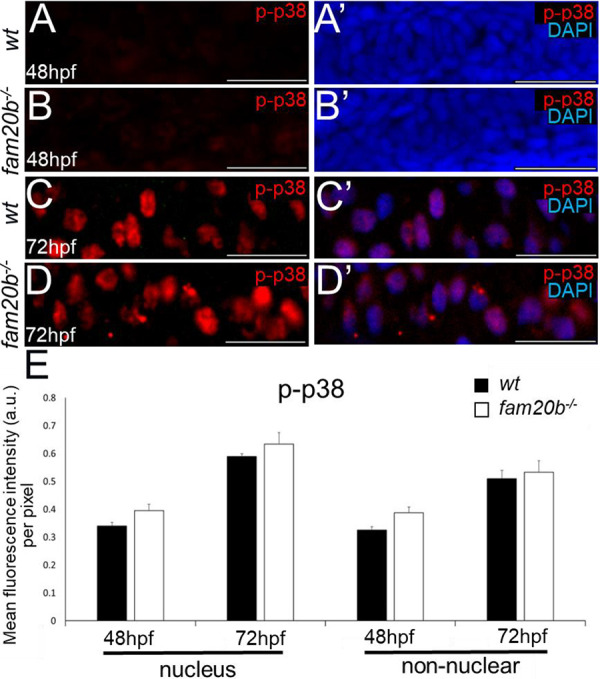
**p38-mediated BMP signalling does not differ in *fam20b^−/−^* chondrocytes.** (A-B′) Condensing ceratohyal mesenchyme of wild types (A,A′) and *fam20b* mutants (B,B′) did not show p-p38 immunoreactivity at 48 hpf. (C-D′) p-p38 immunoreactivity appeared similar in ceratohyal chondrocytes of wild types (C,C′) and *fam20b* mutants (D,D′) at 72 hpf. (E) Quantitative image analyses (*n*=6 for each group) revealed no significant differences in p-p38 levels of *fam20b**^−/−^*** chondrocytes. Scale bars: 50 μm. a.u., arbitrary units; wt, wild type.

To determine whether cartilage PGs inhibit BMP signalling, canonical phospho-Smad1/5/9 (p-Smad1/5/9) and non-canonical phospho-p38 (p-p38) levels were analysed in developing wild-type and *fam20b**^−/−^*** chondrocytes before and after PG secretion. Immunostaining was first confirmed on positive control tissues at 48 hpf; somites were p-Smad1/5/9-positive, and spinal cord was p-p38-positive (Fig. S1A-B′), as reported previously (Patterson et al., 2010; Ji et al., 2002). In condensing ceratohyal mesenchyme at 48 hpf (prior to secretion of cartilage PGs), no clear differences in the levels of p-Smad1/5/9 or p-p38 immunoreactivity were observed between wild types and *fam20b* mutants (Figs 3A-D′, 4A-B′). Indeed, p-p38 did not show detectable immunoreactivity in condensing ceratohyal mesenchyme at 48 hpf (Fig. 4A-B′). Quantitation of p-Smad1/5/9 and p-p38 levels in either the nuclei of cells or non-nuclear regions (i.e. cytoplasm, cell membrane, and intercellular ECM) confirmed these observations, showing no significant changes in ceratohyal mesenchyme of *fam20b* mutants, compared with wild types, before cartilage PGs were secreted (Figs 3K, 4E).

Canonical, but not non-canonical, BMP signalling increased in *fam20b**^−/−^*** ceratohyals when chondrocytes became embedded in cartilage PGs. At 72 hpf, p-Smad1/5/9 immunoreactivity appeared higher in *fam20b**^−/−^*** ceratohyal cells, compared with wild types (Fig. 3E-H′). Quantitation of 72 hpf immunostaining demonstrated that the increase in p-Smad1/5/9 intensity in the nuclei was not significant in ceratohyal cells of *fam20b* mutants compared with wild type, but p-Smad1/5/9 intensity was significantly increased in the non-nuclear regions of *fam20b**^−/−^*** cartilage (Fig. 3K). By 84 hpf, however, p-Smad1/5/9 immunoreactivity was significantly increased in both the nuclei and non-nuclear regions of the ceratohyal in *fam20b* mutants (Fig. 3I-K). This upregulation of BMP signalling in *fam20b**^−/−^*** ceratohyal cells was not specific to subregions of the skeletal element, with all cells apparently having increased p-Smad1/5/9 levels throughout the ceratohyal of *fam20b* mutants. By contrast, non-canonical BMP signalling was unaffected in *fam20b**^−/−^*** ceratohyals when chondrocytes became embedded in PGs, as p-p38 immunostaining was similar in nuclei and non-nuclear regions between wild types and *fam20b* mutants at 72 hpf (Fig. 4C-E).

**Fig. 5.**
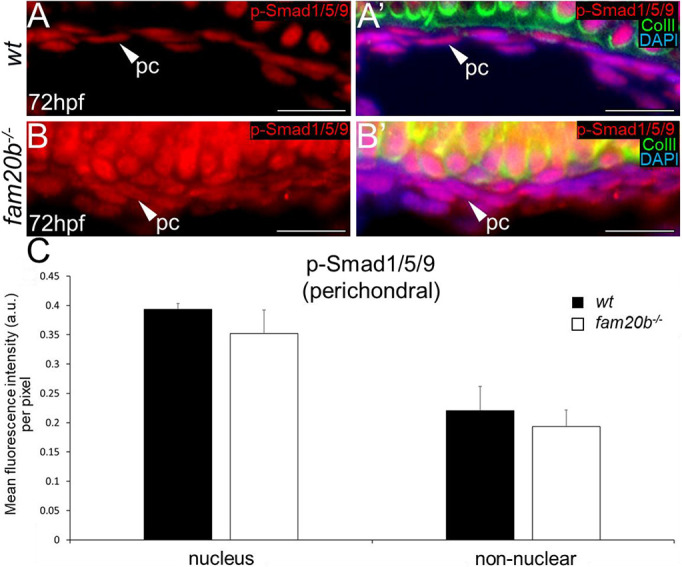
**Smad-mediated BMP signalling does not increase in the *fam20b^−/−^* perichondrium region.** (A-B′) p-Smad1/5/9 immunoreactivity did not show a clear difference in *fam20b^−/−^* perichondral cells (B,B′) at 72 hpf, compared with wild types (A,A′). (C) Quantitative image analyses (*n*=3 for each group) revealed no significant differences in p-Smad1/5/9 levels of *fam20b^−/−^* perichondral cells. Scale bars: 50 μm. a.u., arbitrary units; ColII, Collagen type II; pc, perichondral cells; wt, wild type.

Mutant *fam20b* zebrafish form perichondral bone earlier than wild types, and BMPs can stimulate osteoblast differentiation (Lian et al., 2006; Eames et al., 2011; Yamaguchi et al., 2000). To evaluate whether increased BMP signalling directly induced perichondral bone in *fam20b****^−/−^*** ceratohyals, p-Smad1/5/9 levels were evaluated in perichondral cells, which were identified as more flattened cells that were external to Col2-positive cartilage matrix. At 72 hpf, no differences in p-Smad1/5/9 levels were observed or quantitated in either nuclei or non-nuclear regions of *fam20b****^−/−^*** perichondral cells, compared with wild types (Fig. 5). In total, these results suggest that increased canonical BMP signalling in chondrocytes specifically drives early endochondral ossification in *fam20b****^−/−^*** zebrafish.

### Inhibiting BMP signalling in wild types delays cartilage maturation and perichondral bone formation

**Fig. 6.**
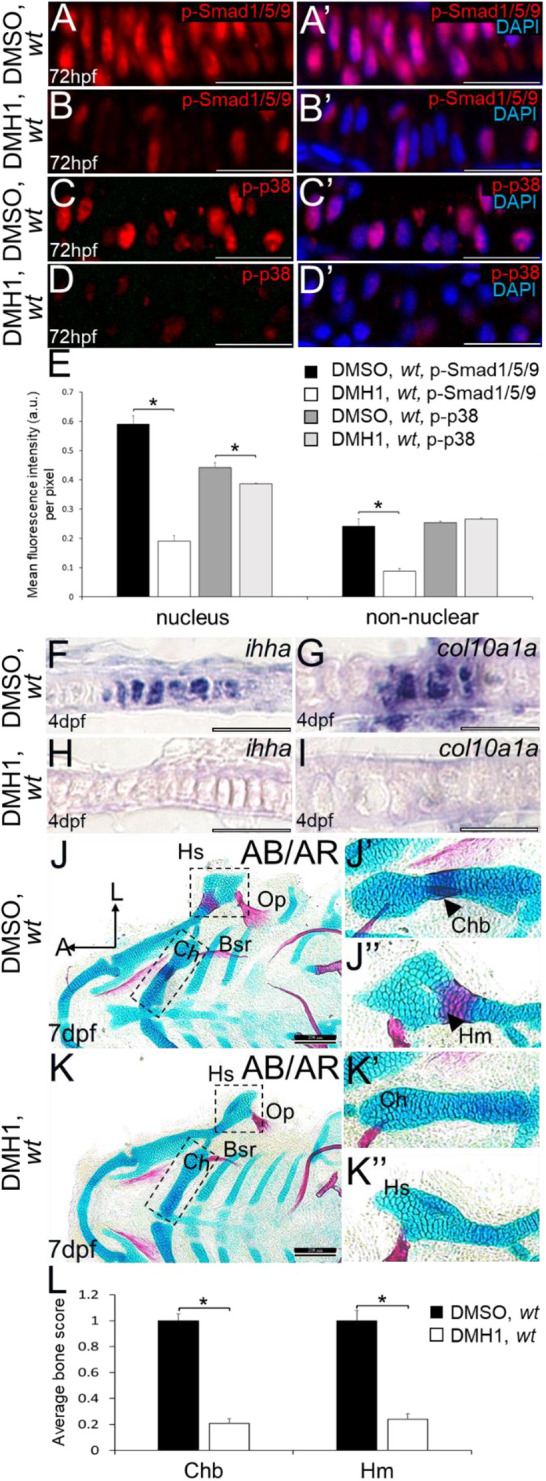
**Inhibition of BMP signalling by DMH1 in wild types reduces cartilage maturation gene expression and perichondral bone formation.** (A-D′) Immunostaining of wild-type chondrocytes at 72 hpf (i.e. after 24 h of treatment) demonstrated that DMH1 treatment reduced p-Smad1/5/9 (B,B′) and p-p38 (D,D′) immunoreactivity, compared with DMSO-treated control chondrocytes (A,A′,C,C′). (E) Quantitative image analyses (*n*=3 for each group) revealed significant decreases in p-Smad1/5/9 and p-p38 levels in nuclei, as well as a significant decrease in p-Smad1/5/9 in non-nuclear regions, of DMH1-treated chondrocytes, compared with DMSO-treated control chondrocytes (*P<0.05, one-way ANOVA and paired Student's *t*-test). (F-I) Compared with DMSO-treated control chondrocytes (F,G), DMH1 treatment decreased expression of the chondrocyte maturation genes *ihha* and *col10a1a* at 4 dpf (i.e. after 2 days of treatment; H,I). These are representative images from at least 12 samples for each group (at least six samples each from two clutches). (J-K″) Compared with DMSO-treated controls (J-J″), DMH1 treatment appeared to decrease perichondral bone at 7 dpf (i.e. 3 days after treatment ended; K-K″). (L) Quantitation of 100 larvae (20 larvae each from five clutches) for each experimental group confirmed a significant decrease in perichondral bone in DMH-treated wild types (*P<0.05, one-way ANOVA and paired Student's *t*-test). Scale bars: 50 μm (A-D′,F-I); 200 μm (J,K). A, anterior; AB/AR, Alcian Blue/Alizarin Red; a.u., arbitrary units; Bsr, branchiostegal ray; Ch, ceratohyal cartilage; Chb, ceratohyal bone; Hm, hyomandibular bone; Hs, hyosymplectic cartilage; L, lateral; Op, opercle; wt, wild type.

To determine whether changes in BMP signalling can affect the timing of endochondral ossification, wild-type zebrafish embryos were treated with 10 μM DMH1 in DMSO for 48 h starting at 48 hpf. At this time, mesenchymal condensation of specified chondrocytes had already occurred (Fig. 2A-F), allowing a focus on BMP-dependent cartilage maturation without complications from any BMP-dependent effects on earlier stages (Yi et al., 2000). DMH1 treatment appeared to inhibit both canonical and non-canonical BMP signalling in chondrocytes. At 72 hpf (i.e. after 24 h of treatment), DMH1-treated larvae displayed significantly reduced immunoreactivity to both p-Smad1/5/9 and p-p38 in the nuclei of ceratohyal cells, compared with DMSO-treated controls (Fig. 6A-E). The non-nuclear regions of DMH1-treated ceratohyals also showed significantly lower p-Smad1/5/9 staining at 72 hpf (i.e. after 24 h of treatment), whereas p-p38 staining in these regions was unaffected (Fig. 6A-E).

Molecular and histological markers demonstrated a delay in endochondral ossification after DMH1 treatment. Consistent with data showing that BMPs promote cartilage maturation (Volk et al., 2000; Steinert et al., 2009; Zhang et al., 2003; Shum et al., 2004; Seki and Hata, 2004), *ihha* and *col10a1a* expression seemed dramatically reduced in chondrocytes of DMH1-treated zebrafish at 4 dpf (i.e. after 2 days of treatment), compared with DMSO-treated controls (Fig. 6F-I). The amount of perichondral bone, such as seen in either the ceratohyal or hyomandibular, appeared reduced in the craniofacial skeleton of DMH1-treated larvae at 7 dpf (i.e. 3 days after treatment ended), compared with DMSO-treated controls (Fig. 6J-K″). Specificity of the effects of this particular DMH1 treatment protocol on endochondral ossification was reflected by the lack of apparent effect on bones that form by intramembranous ossification, such as the opercle or branchiostegal rays (Fig. 6J-K″). Using a scoring system for perichondral bone (Fig. S2), DMH1-treated larvae had significantly less bone in both the ceratohyal and the hyomandibular, compared with DMSO-treated controls (Fig. 6L). This significant decrease in perichondral bone was confirmed using another quantitative measure of perichondral bone area from Alizarin Red-stained fluorescent images (Fig. S3).

**Fig. 7.**
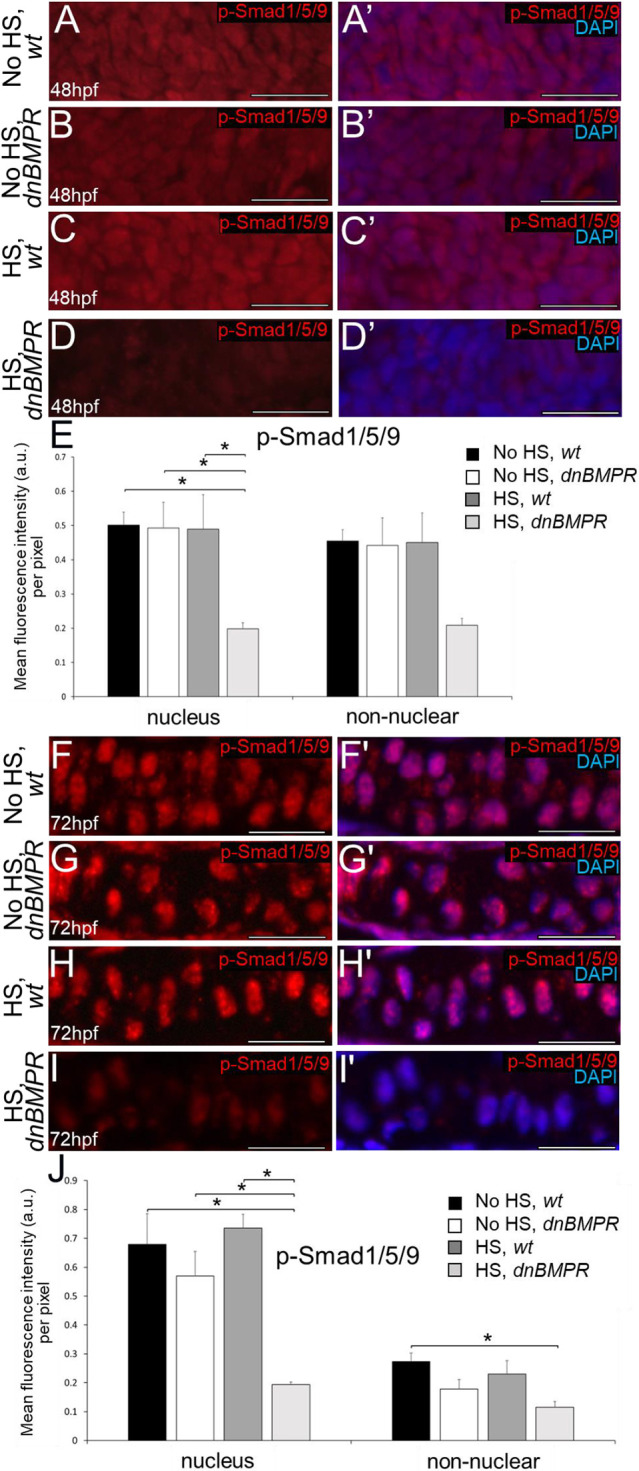
**Smad-mediated BMP signalling is decreased in chondrocytes of heat-shocked, *dnBMPR* zebrafish embryos.** (A-D′) Condensing ceratohyal mesenchyme of heat-shocked *dnBMPR* zebrafish embryos (D,D′) showed reduced levels of p-Smad1/5/9 immunoreactivity at 48 hpf (i.e. 24 after 20-min heat shock), compared with non heat-shocked wild-type (A,A′) and *dnBMPR* (B,B′) embryos and heat-shocked wild-type embryos (C,C′). (E) Quantitative image analyses (*n*=3 for each group) revealed significant decreases in p-Smad1/5/9 levels in nuclei of condensing ceratohyal mesenchyme of heat-shocked *dnBMPR* zebrafish, compared with each control group (*P<0.05, one-way ANOVA and paired Student's *t*-test). (F-I′) Chondrocytes of heat-shocked *dnBMPR* zebrafish embryos (I,I′) showed reduced levels of p-Smad1/5/9 immunoreactivity at 72 hpf (i.e. 48 h after 20-min heat shock), compared with control groups (F-H′). (J) Quantitative image analyses (*n*=3 for each group) revealed significant decreases in p-Smad1/5/9 levels in nuclei, but not in non-nuclear regions, of chondrocytes of heat-shocked *dnBMPR* zebrafish at 72 hpf, compared with each control group (*P<0.05, one-way ANOVA and paired Student's *t*-test). Scale bars: 50 μm. a.u., arbitrary units; HS, heat-shocked; wt, wild type.

**Fig. 8.**
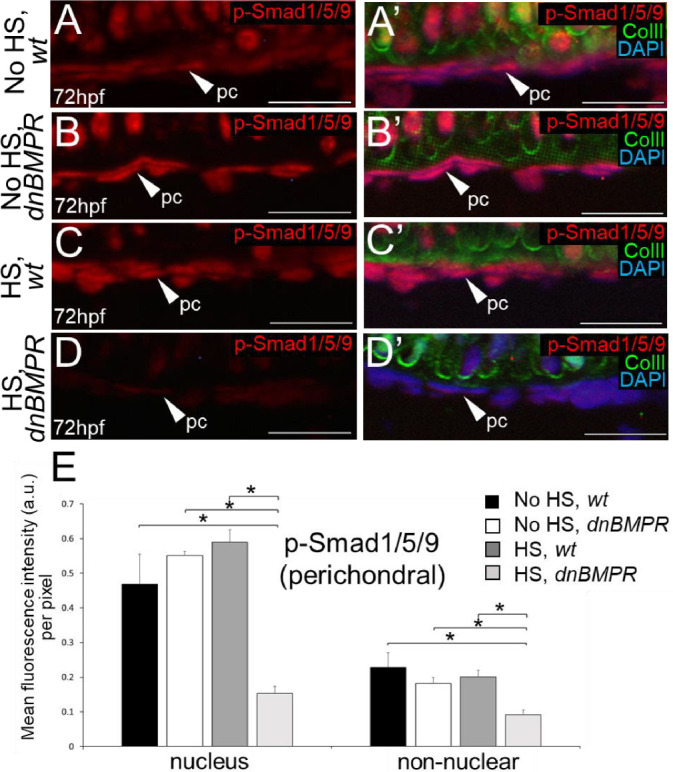
**Smad-mediated BMP signalling is decreased in the perichondrium region of heat-shocked, *dnBMPR* zebrafish embryos.** (A-D′) Levels of p-Smad1/5/9 immunoreactivity appeared reduced in perichondral cells of heat-shocked *dnBMPR* ceratohyals at 72 hpf (i.e. 48 h after 20-min heat shock). (E) Quantitative image analyses (*n*=3 for each group) revealed significant decreases in p-Smad1/5/9 levels in both nuclei and non-nuclear regions of perichondral cells in heat-shocked *dnBMPR* zebrafish, compared with each control group (*P<0.05, one-way ANOVA and paired Student's *t*-test). Scale bars: 50 μm. a.u., arbitrary units; ColII, Collagen type II; HS, heat-shocked; pc, perichondral cells; wt, wild type.

**Fig. 9.**
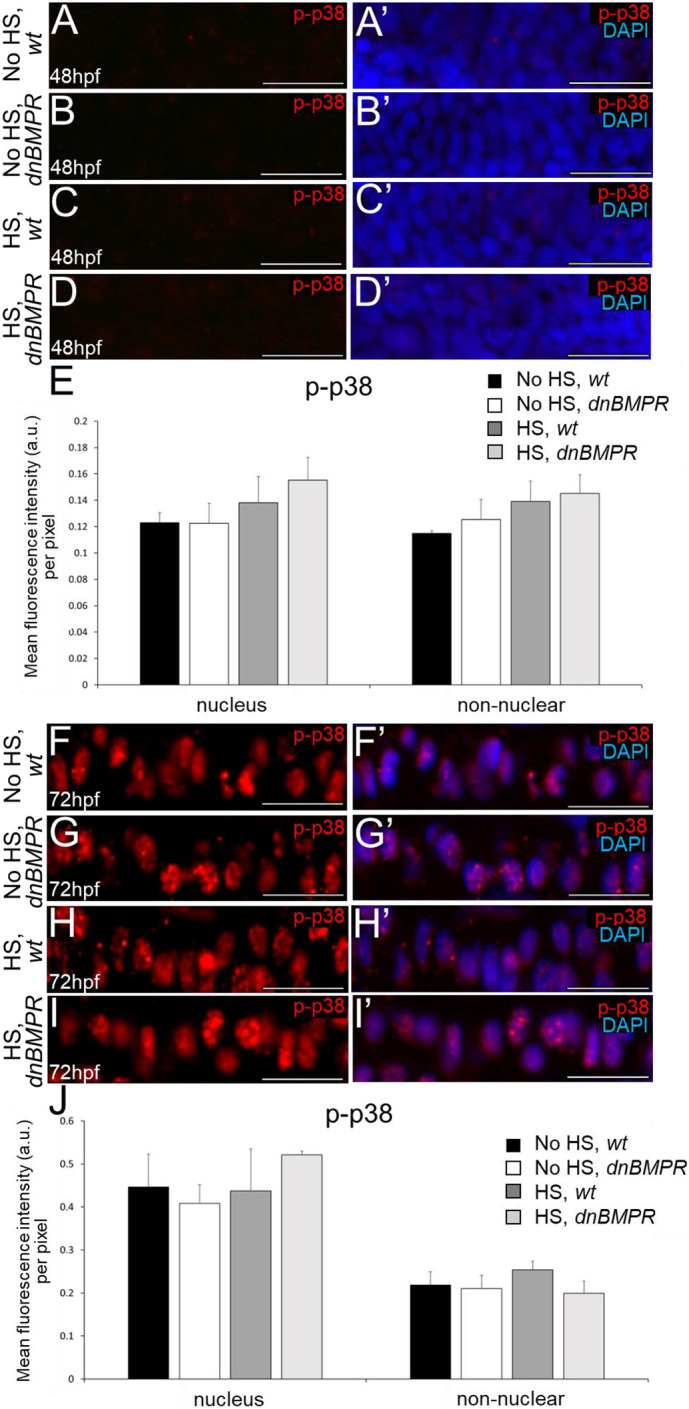
**p38-mediated BMP signalling does not differ in chondrocytes of heat-shocked, *dnBMPR* zebrafish embryos.** (A-D′) Condensing ceratohyal mesenchyme did not show much p-p38 immunoreactivity at 48 hpf in non-heat-shocked wild-type (A,A′) and *dnBMPR* (B,B′) embryos and heat-shocked wild-type (C,C′) and *dnBMPR* (D,D′) embryos (i.e. 24 h after 20-min heat shock). (E) Quantitative image analyses (*n*=3 for each group) revealed no significant differences in p-p38 levels in condensing ceratohyal mesenchyme among experimental groups. One-way ANOVA and paired Student's *t*-test. (F-I′) Chondrocytes of heat-shocked *dnBMPR* zebrafish embryos (I,I′) showed similar levels of p-p38 immunoreactivity at 72 hpf (i.e. 48 h after heat shock) as control groups (F-H′). (J) Quantitative image analyses (*n*=3 for each group) revealed no significant differences in p-p38 levels in chondrocytes of heat-shocked *dnBMPR* zebrafish, compared with each control group. One-way ANOVA and paired Student's *t*-test. Scale bars: 50 μm. a.u., arbitrary units; HS, heat-shocked; wt, wild type.

The effect of BMP signalling on the timing of endochondral ossification was further analysed using a dominant-negative BMP receptor transgenic line under the control of a heat shock promoter (*dnBMPR*; Pyati et al., 2005). Because the receptor transgene is fused to GFP, fluorescent microscopy verified transgene activation within 4 h after a 20-min heat shock at 24 hpf in heterozygous *dnBMPR* zebrafish, but not in controls, including non-heat-shocked *dnBMPR* larvae (Fig. S4A-H). Fluorescence was maintained 24 h after heat shock, but appeared reduced 48 h after the heat shock (Fig. S4I-X), suggesting that the reduction in BMP signalling in heat-shocked *dnBMPR* embryos might be transient, similar to the DMH1 treatment protocol above. Significantly reduced p-Smad1/5/9 immunoreactivity was observed in nuclei of ceratohyal cells of heat-shocked *dnBMPR* zebrafish at both 48 hpf and 72 hpf (i.e. 24 and 48 h after 20-min heat shock), compared with all other control groups (non-heat-shocked wild-type and *dnBMPR* embryos, and heat-shocked wild-type embryos; Fig. 7). Non-nuclear regions of the ceratohyal appeared to have decreased p-Smad1/5/9 immunostaining in heat-shocked *dnBMPR* zebrafish at 48 hpf and 72 hpf, but this was not statistically different compared with all other control groups (Fig. 7). Perichondral p-Smad1/5/9 signalling also decreased significantly in nuclei and non-nuclear regions of heat-shocked *dnBMPR* zebrafish at 72 hpf (i.e. 48 h after heat shock), compared with all other control groups (Fig. 8). In contrast to DMH1 experiments, no significant differences in the levels of p-p38 immunoreactivity were observed in ceratohyals of heat-shocked *dnBMPR* zebrafish at 48 hpf and 72 hpf (i.e. 24 and 48 h after heat shock; Fig. 9). However, both p-Smad1/5/9 and p-p38 immunoreactivity were reduced at 48 hpf in somites and spinal cords, respectively, of heat-shocked *dnBMPR* zebrafish (Fig. S1C-H).

**Fig. 10.**
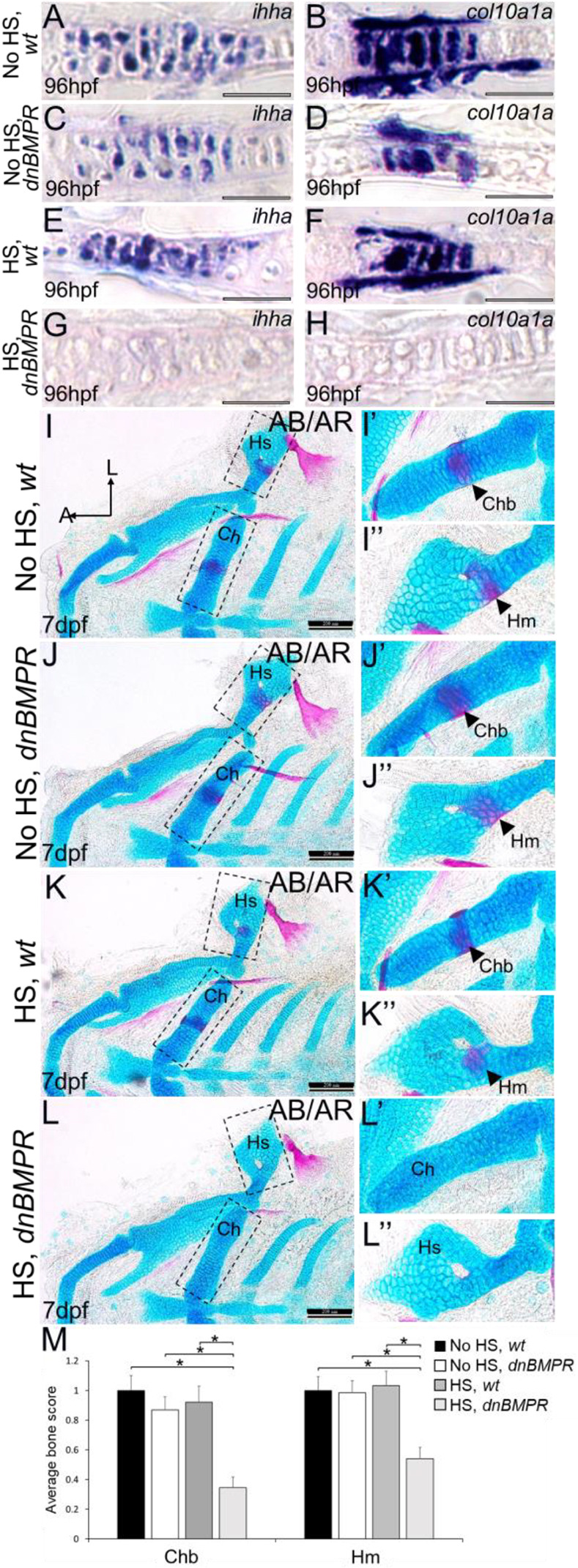
**Inhibition of BMP signalling by heat shocking *dnBMPR* zebrafish embryos reduces cartilage maturation gene expression and perichondral bone formation.** (A-H) Compared with chondrocytes in non-heat-shocked wild-type (A,B) and *dnBMPR* (C,D) embryos and heat-shocked wild-type (E,F) embryos, heat-shocked *dnBMPR* (G,H) chondrocytes had decreased expression of the chondrocyte maturation genes *ihha* and *col10a1a* at 4 dpf (i.e. 3 days after heat shock). These are representative images from at least 12 samples for each group (at least six samples each from two clutches). (I-L″) Compared with control groups (I-K″), perichondral bone appeared to decrease in heat-shocked *dnBMPR* embryos (L-L″) at 7 dpf (i.e. 6 days after heat shock). (M) Quantitation of 100 embryos (20 embryos each from five clutches) for each experimental group confirmed a significant decrease in perichondral bone in heat-shocked *dnBMPR* embryos (*P<0.05, one-way ANOVA and paired Student's *t*-test). Scale bars: 50 μm (A-H); 200 μm (I-L″). A, anterior; AB/AR, Alcian Blue/Alizarin Red; Ch, ceratohyal cartilage; Chb, ceratohyal bone; Hm, hyomandibular bone; Hs, hyosymplectic cartilage; HS, heat-shocked; L, lateral; wt, wild type.

Molecular and histological markers demonstrated a delay in endochondral ossification after *dnBMPR* activation. In heat-shocked *dnBMPR* embryos at 4 dpf (i.e. 3 days after heat shock), *ihha* and *col10a1a* expression was much lower in ceratohyal chondrocytes, compared with all other control groups (Fig. 10A-H). Skeletal histology showed comparable amounts of perichondral bone in the ceratohyal and hyomandibular of 7 dpf larvae that were not heat shocked, and heat-shocked larvae that did not possess the *dnBMPR* transgene (Fig. 10I-K″). In contrast, heat-shocked *dnBMPR* larvae had significantly less perichondral bone than all control groups at 7 dpf (i.e. 6 days after heat shock; Fig. 10L-M).

### Inhibiting BMP signalling rescues the early endochondral ossification in *fam20b^−/−^* zebrafish

**Fig. 11.**
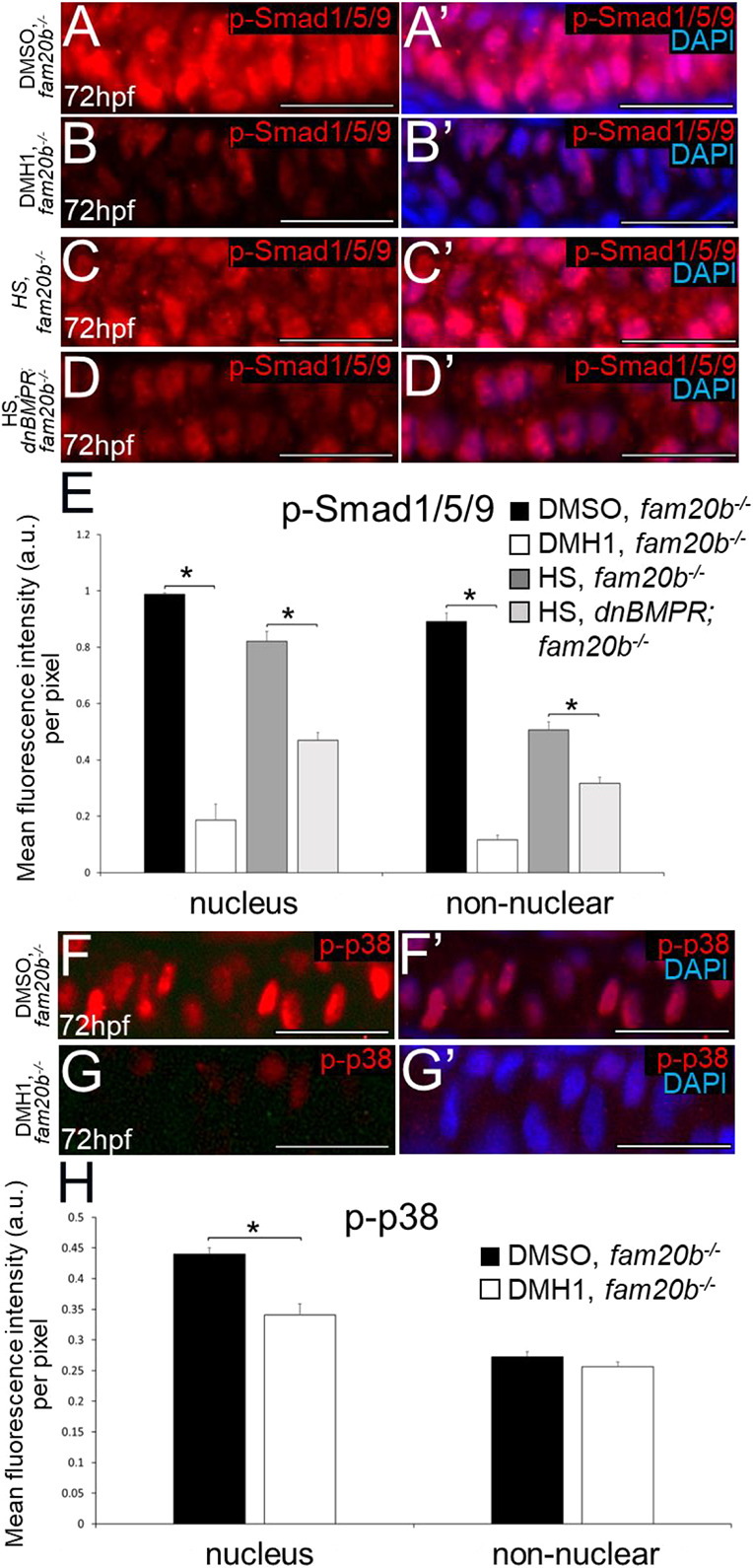
**Inhibition of BMP signalling by DMH1 or by heat shocking *dnBMPR* zebrafish embryos rescues the increased BMP signalling in *fam20b^−/−^* chondrocytes.** (A-D′) Compared with DMSO-treated (A,A′) or heat-shocked (C,C′) *fam20b^−/−^* chondrocytes, levels of p-Smad1/5/9 at 72 hpf appeared decreased in DMH1-treated *fam20b^−/−^* (i.e. after 24 h of treatment; B,B′) or heat-shocked *dnBMPR*; *fam20b^−/−^* (i.e. 2 days after 20-min heat shock; D,D′) chondrocytes. (E) Quantitative image analyses (*n*=3 for each group) revealed significant decreases in p-Smad1/5/9 levels in both nuclei and non-nuclear regions of DMH1-treated or heat-shocked *fam20b^−/−^* chondrocytes, compared with each control group (*P<0.05, one-way ANOVA and paired Student's *t*-test). (F-G′) Compared with DMSO-treated *fam20b^−/−^* chondrocytes (F,F′), levels of p-p38 at 72 hpf (i.e. after 24 h of treatment) appeared decreased in DMH1-treated *fam20b^−/−^* chondrocytes (G,G′). (H) Quantitative image analyses (*n*=3 for each group) revealed significant decreases in p-p38 levels in nuclei of DMH1-treated *fam20b^−/−^* chondrocytes (*P<0.05, one-way ANOVA and paired Student's *t*-test). Scale bars: 50 μm. a.u., arbitrary units; HS, heat-shocked.

To analyse the extent to which increased BMP signalling drives early endochondral ossification in *fam20b* mutants, they were subjected to either DMH1 treatment or *dnBMPR* activation exactly as in the experiments above. Potential treatment of PG-dependent skeletal defects by BMP manipulations has never been reported. Levels of p-Smad1/5/9 immunoreactivity at 72 hpf were significantly decreased in the nuclei and non-nuclear regions of *fam20b****^−/−^*** chondrocytes in both DMH1-treated (i.e. after 24 h of treatment) and heat-shocked *dnBMPR* larvae (i.e. 2 days after 20-min heat shock), compared with control *fam20b* mutants (Fig. 11A-E). DMH1 treatment also significantly decreased p-p38 levels in chondrocyte nuclei, but not non-nuclear regions, at 72 hpf (i.e. after 24 h of treatment), compared with DMSO-treated *fam20b* mutants (Fig. 11F-H).

**Fig. 12.**
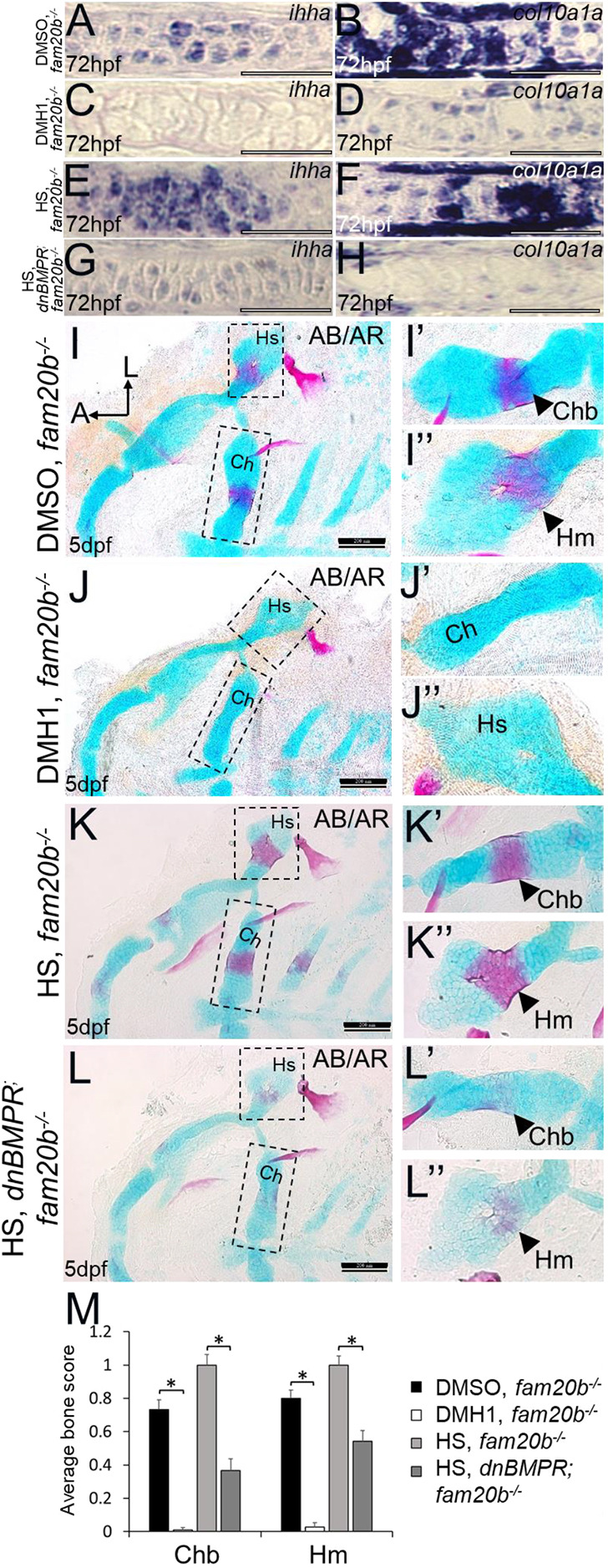
**Inhibition of BMP signalling by DMH1 or by heat shocking *dnBMPR* zebrafish embryos rescues early cartilage maturation gene expression and perichondral bone formation in *fam20b* mutants.** (A-H) Compared with DMSO-treated (A,B) or heat-shocked (E,F) *fam20b^−/−^* chondrocytes, DMH1-treated *fam20b^−/−^* (C,D) or heat-shocked *dnBMPR*; *fam20b^−/−^* (G,H) chondrocytes had decreased expression of the chondrocyte maturation genes *ihha* and *col10a1a* at 72 hpf (i.e. after 24 h of DMH1 treatment, or 2 days after heat shock). These are representative images from at least 12 samples for each group (at least six samples each from two clutches). (I-L″) Compared with respective control groups (I-I″,K-K″), perichondral bone appeared to decrease in DMH1-treated *fam20b^−/−^* (i.e. 1 day after end of 48 h treatment; J-J″) or heat-shocked *dnBMPR*; *fam20b^−/−^* (i.e. 4 days after heat shock; L-L″) embryos at 5 dpf. (M) Quantitation of 60 embryos (20 embryos each from three clutches) for each experimental group of the DMH1 experiment and of 80 embryos (20 embryos each from four clutches) for each experiment group of the *dnBMPR* experiment confirmed a significant decrease in perichondral bone of DMH1-treated *fam20b^−/−^* or heat-shocked *dnBMPR*; *fam20b^−/−^* embryos (*P<0.05, one-way ANOVA and paired Student's *t*-test). Scale bars: 50 μm (A-H); 200 μm (I-L″). A, anterior; AB/AR, Alcian Blue/Alizarin Red; Ch, ceratohyal cartilage; Chb, ceratohyal bone; Hm, hyomandibular bone; Hs, hyosymplectic cartilage; HS, heat-shocked; L, lateral.

**Fig. 13.**
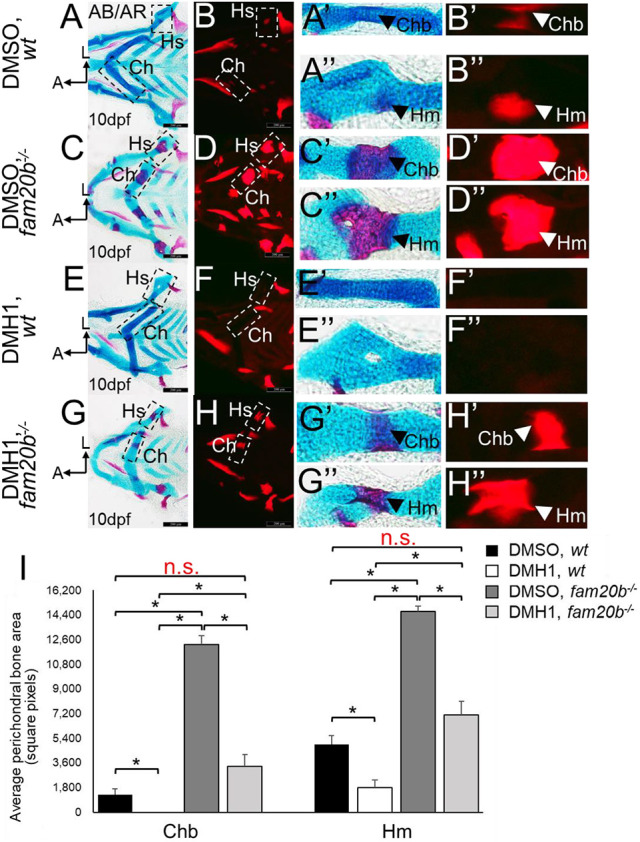
**Inhibition of BMP signalling by early DMH1 treatment reduces perichondral bone formation in *fam20b* mutants down to wild-type levels at 10** **dpf.** (A-H″) Compared with DMSO-treated wild-type controls (A-B″), perichondral bone appeared to decrease in DMH1-treated wild types at 10 dpf (i.e. 6 days after end of 48 h treatment; E-F″). Similarly, perichondral bone at 10 dpf appeared to decrease in DMH1-treated *fam20b* mutants (G-H″), compared with DMSO-treated *fam20b* mutants (C-D″), with levels similar to that seen in DMSO-treated wild types (A-B″). (I) Quantitation of 20 embryos for each experimental group confirmed significant decreases (*P<0.05, one-way ANOVA and paired Student's *t*-test) in perichondral bone of DMH1-treated wild-type or *fam20b^−/−^* embryos. Remarkably, the levels of perichondral bone in DMH1-treated *fam20b* mutants was statistically indistinguishable from DMSO-treated wild-type controls. Scale bars: 200 μm (A-H). A, anterior; AB/AR, Alcian Blue/Alizarin Red; Ch, ceratohyal cartilage; Chb, ceratohyal bone; Hm, hyomandibular bone; Hs, hyosymplectic cartilage; HS, heat-shocked; L, lateral; n.s., not significant; wt, wild type.

Molecular and histological markers demonstrated a decrease in endochondral ossification of *fam20b* mutants after DMH1 treatment or *dnBMPR* activation. Expression of the cartilage maturation genes *ihha* and *col10a1a* was lower in DMH1-treated *fam20b**^−/−^*** ceratohyal chondrocytes at 72 hpf (i.e. after 24 h of treatment), compared with DMSO-treated *fam20b* mutants (Fig. 12A-D). *ihha* and *col10a1a* also showed decreased expression in *fam20b**^−/−^*** chondrocytes of heat-shocked *dnBMPR* larvae at 72 hpf (i.e. 2 days after heat shock), compared with heat-shocked *fam20b* mutant controls (Fig. 12E-H). Analyses of markers that are normally downregulated during cartilage maturation, such as *col2a1a*, *col11a2* and *sox9a*, further confirmed that DMH1 treatment rescued the accelerated cartilage maturation in *fam20b* mutants, as these markers failed to be downregulated at 4 dpf in chondrocytes of DMH1-treated *fam20b* mutants (i.e. after 48 h of treatment), compared with DMSO-treated controls (Fig. S5). Regarding the bone phenotype of *fam20b* mutants, two measures of skeletal histology showed that DMH1 treatment significantly decreased early perichondral bone formation in the *fam20b**^−/−^*** craniofacial skeleton at 5 dpf (i.e. 1 day after end of 48 h treatment), compared with DMSO-treated *fam20b* mutants (Fig. 12I-J″,M; Fig. S6). *fam20b* mutant larvae with an activated *dnBMPR* transgene also had significantly reduced perichondral bone staining at 5 dpf (i.e. 4 days after heat shock), compared with heat-shocked *fam20b* mutant controls (Fig. 12K-M). To gain insight into the nature of the proposed rescue of perichondral bone in *fam20b* mutants, quantitation was also performed at 10 dpf (i.e. 6 days after end of 48 h treatment). At 10dpf, DMH1 treatment significantly reduced perichondral bone in wild types, compared with DMSO-treated controls (Fig. 13A-B″,E-F″). Indeed, DMH1-treated *fam20b* mutant perichondral bone was still significantly decreased, compared with DMSO-treated *fam20b* mutants (Fig. 13C-D″,G-I). Remarkably, the levels of perichondral bone in DMH1-treated *fam20b* mutants at 10 dpf were statistically indistinguishable from DMSO-treated wild-type controls (Fig. 13A-B″,G-I). In total, these data suggest that inhibition of BMP-dependent cartilage maturation rescues the early endochondral ossification in *fam20b* mutants.

## DISCUSSION

Since the discovery in *Drosophila* over 25 years ago that PGs can modulate growth factor signalling (Jackson et al., 1997), PG-rich tissues, such as neural or skeletal tissues, have served as great models to understand how PGs affect signalling in vertebrates (Schwartz and Domowicz, 2023; Pegge et al., 2020; Brown and Eames, 2016). Indeed, many PG mutants have skeletal defects that are associated with changes in growth factor signalling (Klüppel et al., 2005; Cortes et al., 2009; Matsumoto et al., 2010; Inubushi et al., 2017; Fisher et al., 2006; Paine-Saunders et al., 2000; Hilton et al., 2005; Koziel et al., 2004; Eames et al., 2010). To clarify discrepancies in published reports (Kawashima et al., 2020; Klüppel et al., 2005; Lyu et al., 2022), here we analysed how cartilage PGs modulate BMP signalling, additionally providing new, temporally controlled insights into how BMP signalling regulates cartilage maturation during endochondral ossification. In summary, these data support the hypothesis that PGs normally inhibit canonical BMP-dependent cartilage maturation to delay endochondral ossification.

Our findings are in line with recent reports showing that BMP signalling is increased in chondrocytes when the PG synthesis genes *Ext1* or *Chsy1* are mutated (Kawashima et al., 2020; Lyu et al., 2022). However, a decrease in BMP signalling in chondrocytes of *Chst11* mutants (Klüppel et al., 2005) might be caused by the different roles of the mutated genes in PG production. Ext1 and Chsy1 catalyse synthesis of the repeating disaccharides of heparan sulfate (HS) and chondroitin sulfate (CS) PGs, respectively, and Fam20b phosphorylation of the PG linker sugar xylose drives processivity of both HSPGs and CSPGs (Brown and Eames, 2016). By comparison, Chst11 regulates a specific aspect of CSPG sulfation, adding a sulfate to position 4 of the N-acetylgalactosamine (GalNAc) sugar (Klüppel, 2010). PG sulfation plays a major role in binding of growth factors (Cortes et al., 2009; Klüppel et al., 2005; Nadanaka et al., 2008; Schwartz and Domowicz, 2023), but perhaps the specificity of the Chst11 mutation reveals a different role for that sulfation site compared with the broader overall reduction in sulfated PGs resulting from *Ext1*, *Chsy1* or *Fam20b* mutations.

The exact mechanism by which cartilage PGs inhibit BMP signalling is unclear, but our results are consistent with cartilage PGs sequestering BMPs in the ECM. Indeed, all chondrocytes in *fam20b**^−/−^*** ceratohyals showed increased p-Smad1/5/9 levels, suggesting that PG limitation of BMP diffusion is not a major mechanism in zebrafish larval cartilages. Cartilage PG inhibition of BMP signalling might be a conserved trait among vertebrates, based upon similar findings in mouse and now zebrafish, but more animals need to be tested to support this prospect. More broadly, the role of PGs in skeletal development traditionally would be described as a permissive interaction (Gilbert and Barresi, 2000), merely providing an environment that permits instructive cues (e.g. growth factors) to reach their target cells. However, our data argue that at least some biological roles of ECM should no longer be considered permissive interactions. As shown here and discussed below, the levels of PGs can affect the timing of skeletal development in an instructive fashion. In wild types, the levels of cartilage PGs have been calibrated through evolution to regulate growth factor signalling and achieve ‘normal’ timing of skeletal development, whereas in *fam20b* mutants, the levels of cartilage PGs are decreased, and skeletal development is accelerated.

Our data lend firm *in vivo* support to the idea that BMP signalling not only promotes cartilage maturation, but in doing so also regulates the overall timing of endochondral ossification. Cartilage and bone development are tightly linked through signalling molecules, such as Ihh, during endochondral ossification. *Ihh* is expressed specifically in mature chondrocytes, and consistent with *Ihh* being a direct target of BMP signalling (Volk et al., 2000; Seki and Hata, 2004; Zhang et al., 2003; Shum et al., 2004), DMH1 treatment or *dnBMPR* activation reduced *ihha* expression in zebrafish cartilage. The timing of BMP inhibition employed here was crucial to focus on the role of BMP in cartilage maturation, avoiding complications of the earlier role of BMPs in chondrogenesis (Yi et al., 2000). The clearest previous *in vivo* study specifically relating BMP signalling to cartilage maturation used a transgene that appeared to have delayed expression in limb cartilages, so maturation occurred, but was blocked at later time points (Retting et al., 2009). Linking cartilage maturation to perichondral bone, Ihh induces osteoblast differentiation in the perichondrium (St-Jacques et al., 1999; Hammond and Schulte-Merker, 2009; Long et al., 2004; Eames et al., 2011), and inhibition of BMP-dependent *ihha* expression delayed perichondral bone formation in wild types and *fam20b* mutants. Although BMP can also induce osteoblast differentiation directly (Lian et al., 2006; Yamaguchi et al., 2000), the absence of increased levels of p-Smad1/5/9 in the *fam20b* mutant perichondrium argues that BMP-dependent cartilage maturation indirectly drives perichondral bone formation in cartilage PG mutants via Ihh expression. Because global *dnBMPR* activation lowered p-Smad1/5/9 levels in both chondrocytes and perichondral cells, functional experiments that target the perichondrium more specifically, such as perichondrium-specific gene constructs, will be required to address this possibility directly. Nevertheless, these results are the first to demonstrate that skeletal defects in a PG mutant animal model can be treated by modulating BMP signalling. Furthermore, our data on *fam20b* mutants at 10 dpf (6 days after DMH1 treatment ended) suggested that even a short treatment period early in a disease might cause lasting, clinically relevant effects.

Our data reinforce that canonical BMP signalling likely drives cartilage maturation *in vivo* (Retting et al., 2009; Kawashima et al., 2020). Correlating with early *ihha* and *col10a1a* expression, *fam20b* mutants had increased p-Smad1/5/9 in chondrocyte nuclei at 84 hpf. However, levels of p-p38 did not differ in *fam20b* mutant chondrocytes, demonstrating that canonical Smad signalling mediates PG-dependent BMP signalling during cartilage maturation. Interestingly, p-Smad1/5/9 levels appeared to first increase in the cytoplasm of chondrocytes at 72 hpf, but not in the nucleus. Significant increases in p-Smad1/5/9 levels in the nucleus were not observed until slightly later, at 84 hpf. These observations suggest that nuclear translocation of activated Smads is inhibited when decreased PGs allow more BMP signalling to occur in early stages of chondrocyte differentiation. Perhaps Smad1/5/9 have specific inhibitors of nuclear import in this context, similar to what has been proposed regarding Smad2/3 linker phosphorylation or Imp7/8 inhibition (Hill, 2009; Jiang et al., 2015).

Experimental inhibition of BMP signalling showed disparate effects on canonical and non-canonical pathways, serving as a reminder to verify the specificity of techniques employed in the cell type(s) of interest. Whereas DMH1 treatment reduced both p-Smad1/5/9 and p-p38 immunoreactivity in chondrocytes, activation of a dominant-negative BMPRIA/ALK3 (i.e. *dnBMPR*) only lowered chondrocyte p-Smad1/5/9 levels. This *dnBMPR* result even differed in the spinal cord of the same fish, where p-p38 levels also decreased. Cell type-specific combinations of ligands and the ligand specificity of BMPRIs might explain these differences, because different ligands might drive different responses in different cell types. DMH1 is thought to specifically antagonize the intracellular kinase domain of the BMPRI ALK2, which can bind both BMPs and activins, and BMPRIA is relatively specific to BMP ligands (Gomez–Puerto et al., 2019; Hao et al., 2010). In chondrocytes, perhaps ALK2 is responding along both canonical and non-canonical pathways (Cocolakis et al., 2001), whereas BMPR1A only signals canonically. In spinal cord, maybe BMPR1A signals through the non-canonical pathway. Accordingly, ligand- and cell type-specific responses need to be evaluated in studies of BMP signalling.

In summary, we show that cartilage PGs normally delay endochondral ossification via inhibition of canonical BMP-dependent cartilage maturation. Furthermore, we provide the first data demonstrating a rescue of skeletal defects in a PG mutant through BMP signalling modulation. In addition to improving our understanding of normal developmental processes involving BMP signalling, these findings put further emphasis on growth factor therapies for a variety of PG-dependent skeletal anomalies. Osteoarthritis (OA), for example, is the most common skeletal defect, especially in a rapidly aging human population. Articular cartilage in OA is characterized by a similar phenotype as seen in some PG mutant models, including loss of sulfated PGs in cartilage, ectopic cartilage maturation, and associated extra perichondral ossification (osteophytes; Kawashima et al., 2020, Domowicz et al., 2009, Eames et al., 2011, Pitsillides and Beier, 2011, Mis et al., 2014). If loss of sulfated PGs is the primary defect in OA, but inducing PG synthesis and sulfation is unfeasible therapeutically, then these animal models suggest that OA progression could be blocked by using growth factor therapies to inhibit cartilage maturation. Indeed, studies in experimental OA models have shown varying success by targeting TGFβ signalling (van der Kraan, 2022). Other growth factor pathway therapies are even currently in clinical trials, but, although some are related to cartilage maturation, most of these pathways are associated with inflammation and pain (Shentu et al., 2022).

## MATERIALS AND METHODS

### Zebrafish lines and sample preparation

All fish lines and embryos were maintained at the University of Saskatchewan according to established protocols (Westerfield, 1995) with University Animal Care Committee approval. Zebrafish lines used were wild type AB, *fam20b^b1127^* (Eames et al., 2011) and *Tg(hsp70l:dnBmpr1a-GFP)w30* (Pyati et al., 2005). PCR genotyping was carried out for *fam20b* mutants as described (Eames et al., 2011), and for *dnBMPR* transgenics using forward and reverse primers 5′-CGTGCTGAAGTCAAGTTTGAAGGTG-3′ and 5′-CCATGCCATGTGTAATCCCAGC-3′, respectively. Unless otherwise noted, all samples were fixed on a rocker overnight at 4°C in 4% paraformaldehyde in PBS, followed by dehydration in an ethanol series. For cryosectioning, samples were washed through an OCT/30% sucrose series, embedded in OCT (Tissue-Tek; Sakura Finetek USA), and cut at 7 µm using a Microm cryostat from ESBE Scientific.

### Fam20b biochemical assays

To enable Fam20b secretion, cDNA fragments of truncated forms of wild-type, *fam20b ^1125b^* and *fam20b ^1127b^*, lacking the first 29 N-terminal amino acids, were amplified by PCR from bact2-fam20b.wt-polyA, bact2-fam20b.b1125-polyA and bact2-fam20b.b1127-polyA plasmids (Eames et al., 2011) as templates, respectively, using a forward primer (5′-GAAGATCTGGATCAGCCGCTAGCCGC-3′) containing an in-frame BglII site and a reverse primer (5′-GAAGATCTGGAAAAAACCTCCCACAC-3′) containing a BglII site located 141 bp downstream of the stop codon. PCR was carried out with KOD-Plus DNA polymerase (Toyobo) for 30 cycles at 94°C for 30 s, 58°C for 30 s and 68°C for 120 s in 5% (v/v) DMSO. Each PCR fragment was subcloned into the BamHI site of pGIR201protA (Kitagawa and Paulson, 1994), resulting in the fusion of the insulin signal sequence and the Protein A sequence present in the vector (Izumikawa et al., 2008).

Each expression plasmid (6.0 μg), along with the negative, empty-vector control, was transfected into COS-1 cells on 100-mm-diameter plates using FuGENE 6 (Promega), according to the manufacturer's instructions. At 2 days after transfection, 1 ml of the culture medium was collected and incubated with 10 μl of IgG–Sepharose (Cytiva) for 1 h at 4°C. The beads recovered by centrifugation at 150 ***g*** for 2 mins were washed with and then resuspended in the assay buffer (Cytiva). To normalize for the amount of recovered protein, proteins were subjected to SDS-PAGE followed by western blotting with IgG antibody (Cytiva) and ECL select detection reagent (Cytiva). Blotting images were obtained and normalized with a luminescent image analyser Image Quant 4000 and Image Quant TL, respectively. Subsequent testing for kinase activity of purified proteins was carried out using Galb1-4Xylβ1-O-ITI as an acceptor, as described previously (Koike et al., 2022).

### Laser capture microdissection and RNA sequencing

RNA was isolated using laser capture microdissection of tissue sections of cranial cartilage in the occipital region of wild-type 6 dpf zebrafish larvae, amplified, and sequenced as described previously (Gomez-Picos et al., 2022; Nguyen et al., 2023). Three independent samples were processed, and normalized read counts were determined as described previously (Gomez-Picos et al., 2022; Nguyen et al., 2023).

### Experimental inhibition of BMP signalling *in vivo*

Treatments with DMH1, a selective chemical inhibitor of type 1 BMP receptors shown to work in zebrafish (Hao et al., 2010), were performed on 2 dpf zebrafish embryos, because treating at earlier time points caused morphological changes to cartilage elements, likely as a result of earlier roles for BMP (Fig. S7). Wild-type zebrafish embryos were treated with 3 μM, 10 μM and 30 μM DMH1 (Selleckchem) or an equivalent volume of DMSO as a solvent control in embryo medium (EM; Westerfield, 1995) for 48 h before it was replaced with regular EM. For further experiments, 10 μM was chosen based on reproducible effects on the skeleton with minimal embryonic deaths and deformations (Fig. S8).

To activate *dnBMPR* expression, embryos at 1 dpf were heat shocked in 50 ml Falcon tubes filled with 30 ml EM for 20 min in a 40°C water bath. Many alternative heat-shock strategies were tested, including once at 2 dpf, 3 dpf and 4 dpf, or multiple heat shocks on successive days. One heat shock at 1 dpf was chosen for further experiments, based on reproducible effects on the skeleton with minimal embryonic deaths and deformations (data not shown). In addition to PCR genotyping, *dnBMPR* embryos were identified by GFP expression after heat shock using a Leica M205 microscope.

### Immunofluorescence and image quantitation

As published (Eames et al., 2010), whole zebrafish or cryosections were digested in 0.1% Trypsin (MP Biomedicals)/1 mM EDTA/1× PBS for 30 min at 37°C and then 0.5% hyaluronidase (Worthington Biochemical Corporation)/PBST (1×PBS +0.1% Triton X-100) for 30 min at 37°C for antigen retrieval. Primary rabbit antibodies for p-Smad1/5/9 (called p-Smad 1/5/8, sc-6031-R, Santa Cruz Biotechnology; AB3848-I, Millipore), p-p38 MAPK (4511, Cell Signalling Technology) and mouse anti-COL II (II-III6B3-s, Developmental Studies Hybridoma Bank) were applied at 1:100 overnight at 4°C, and then secondary Alexa Fluor 594 goat anti-rabbit IgG and Alexa Fluor 448 goat anti-mouse IgG (A11012 and A32723, Thermo Fisher Scientific) were applied at 1:1000 for 4 h at room temperature.

For quantitation of antibody staining in nuclei and non-nuclear regions of cryosections, images were captured by a Leica DFC550 camera attached to a Nikon Eclipse E600 fluorescent microscope, using the same camera settings for all experimental groups in a given time point. Analysed with ImageJ software (http://rsbweb.nih.gov/ij/), DAPI images were thresholded to define the nuclear region pixels, and inverse selection defined the non-nuclear region. Mean pixel intensity of immunostaining was measured separately for the nuclear and non-nuclear regions. To normalize for any differences in cell numbers in the section images, the mean pixel intensity of the nuclear or non-nuclear region was divided by number of DAPI-thresholded pixels. Three samples were analysed in each group.

### *In situ* hybridization

RNA *in situ* hybridization was performed on thawed cryosections using RNA probes for *col10a1a* and *ihha* (Eames et al., 2011), diluted in hybridization buffer (1.3×SSC, 50% formamide, 10% dextran sulfate, 1 mg/ml rRNA, 1× Denhardt's) at a concentration of 1 mg/ml, denatured for 5 mins at 70°C, and then loaded onto sections to hybridize overnight while cover-slipped at 70°C. After one 15-min and three 30-min washes at 70°C in washing solution (1×SSC, 50% formamide, 0.1% Tween20), slides underwent three 30-min washes in 1×MABT at room temperature. After 2-3 h in blocking solution (1×MABT, 20% sheep serum, 2% Boehringer Blocking Reagent), slides were incubated overnight in a humidity chamber at room temperature with blocking solution containing 1:5000 anti-DIG alkaline phosphatase (AP) antibody (11093274910, Roche, Sigma-Aldrich). After five 20-min washes in 1×MABT at room temperature and two 10-min washes with AP buffer [100 mM NaCl, 50 mM MgCl_2_, 100 mM Tris (pH 9.5), 0.1% Tween-20], slides were stained with 3.5 µl/ml NBT (Sigma-Aldrich) and 2.6 µl/ml BCIP (Sigma-Aldrich) in AP buffer with 8% polyvinyl alcohol (Sigma-Aldrich) overnight at 37°C. Once the desired signal was achieved, slides were washed with PBST twice for 5 mins each, then ddH_2_O for 5 mins, twice. Sections were dehydrated through graded fashion to 100% ethanol, cleared in xylene, and mounted for imaging.

### Histological staining and bone quantitation

Safranin O/Fast Green staining on frozen sections was performed as previously described (McManus and Mowry, 1960). Whole-mount zebrafish embryos were stained with an acid-free Alcian Blue and Alizarin Red protocol, as previously published (Eames et al., 2011). To quantitate results, stained perichondral bones were scored on a point system of 0, 1, 2 or 3 (Fig. S2). Several clutches of embryos were scored in this manner. Lower jaws with supporting posterior skeleton were dissected from representative fish in each clutch, mounted on glass slides, and imaged using bright-field microscopy. Alternatively, fluorescent images of the Alizarin Red staining were captured and the number of pixels of perichondral bone was quantitated using ImageJ.

### Statistical analyses

Quantitative data for each experimental group were subjected to one-way ANOVA and paired Student's *t*-test analyses using IBM SPSS version 28.0 software, and *P*<0.05 was considered statistically significant. Error bars represent s.e.m.

## Supplementary Material

10.1242/develop.201716_sup1 Supplementary information
