# Supplementary material for: Proteoglycan inhibition of canonical BMP-dependent cartilage maturation delays endochondral ossification
Source: Development. 2024 Jan 12;151(2):dev201716. doi: 10.1242/dev.201716 (PMC10820745; doi:10.1242/dev.201716)
Supplement: Supplementary information [file develop-151-201716-s1.pdf]

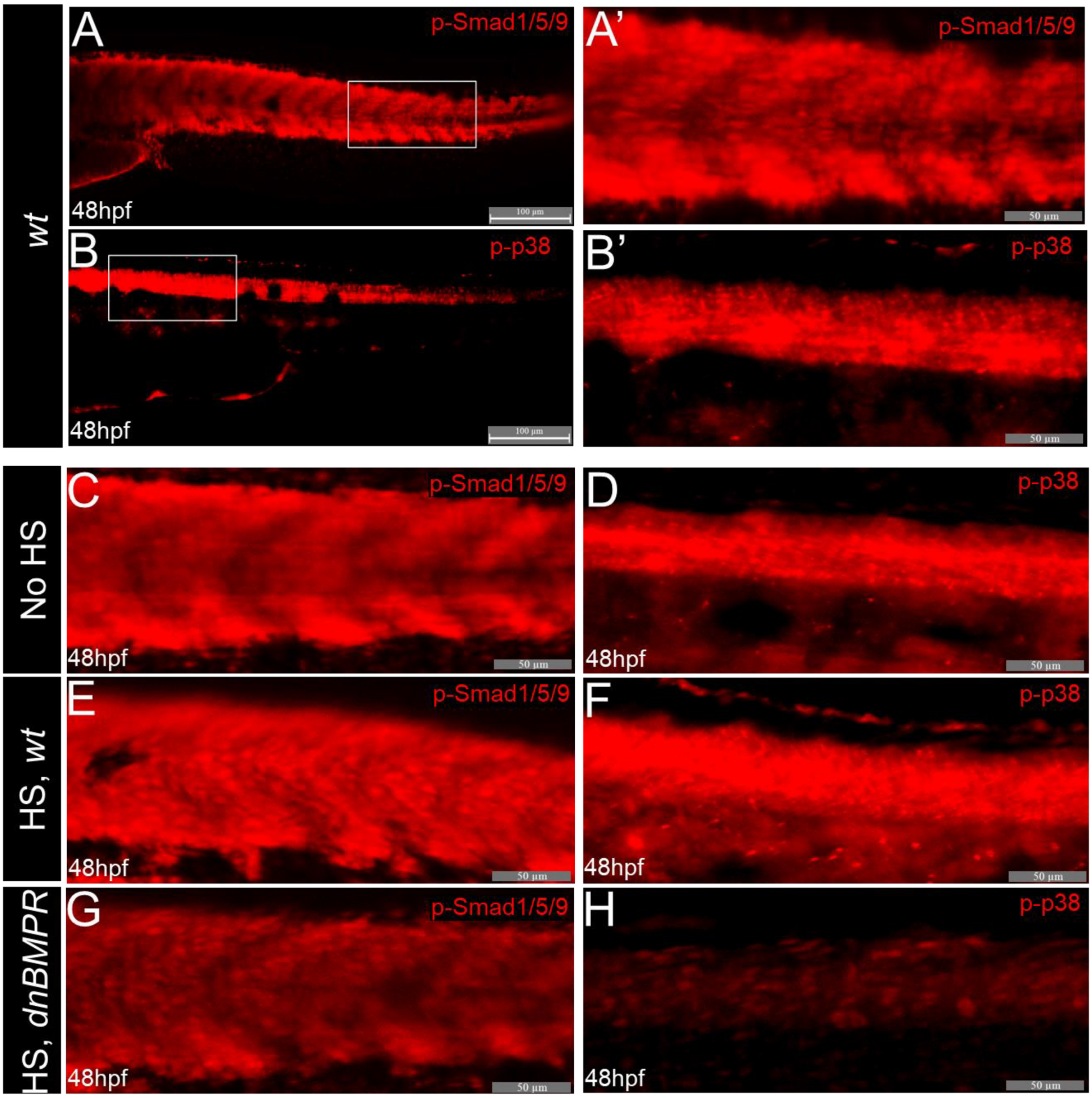

Fig. S1. *dnBMPR* transgene expression inhibits BMP signalling in somites and spinal cord. (A-B') Whole mount immunostaining demonstrated the expression of p-Smad1/5/9 and p-p38 in somite (A,A') and spinal cord (B,B') of zebrafish embryos at 48hpf. **(C-H)** *dnBMPR* transgene activation by heat shock decreased p-Smad1/5/9 and p-p38 immunoreactivity in somite and spinal cord (i.e., 24 hours after heat shock). Scale bars: A-B'=100μm; C-H=50μm. Abbreviations: HS=heat-shocked.

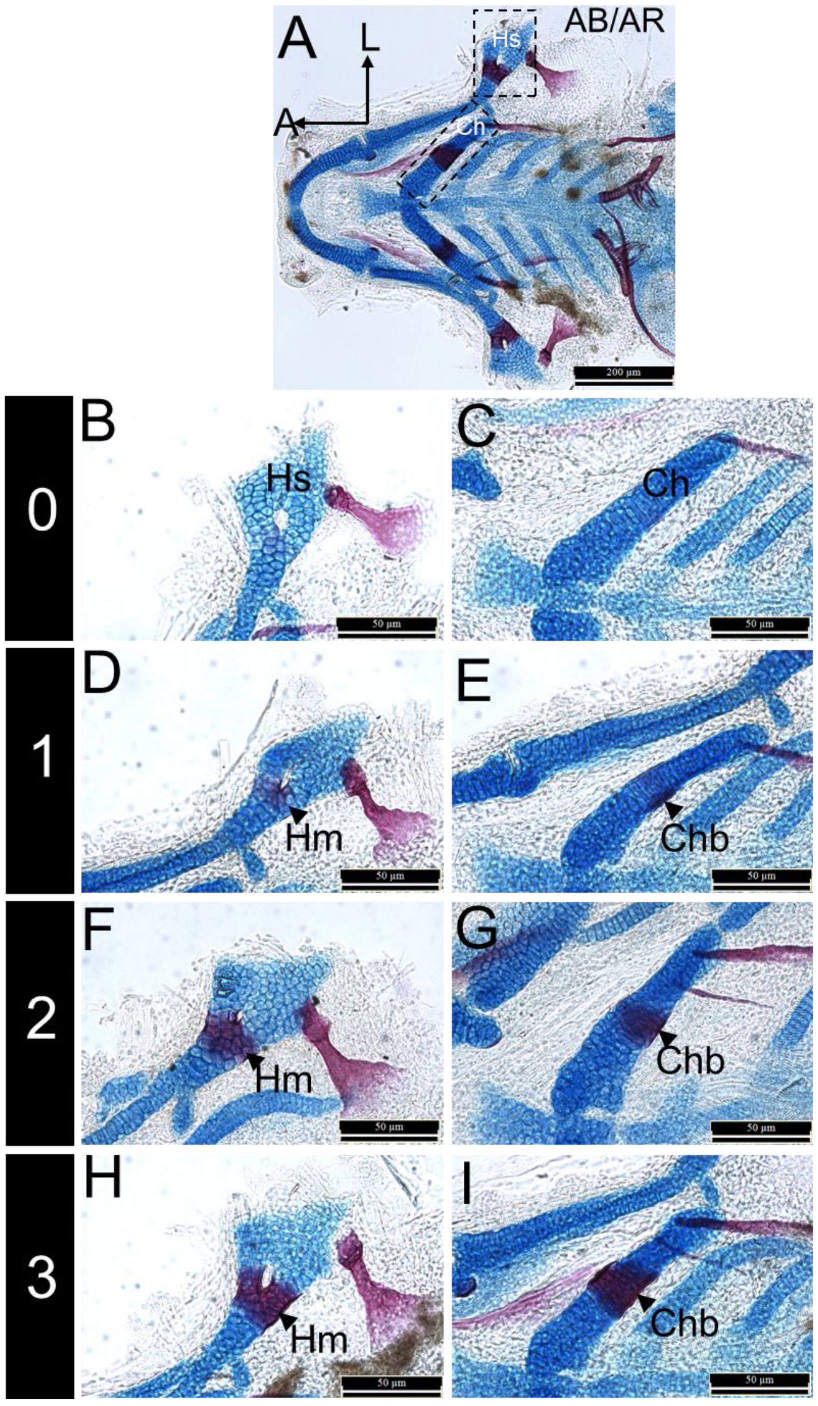

Fig. S2. Skeletal histology scoring standards. (A) Pharyngeal larval zebrafish skeletons were dissected and flat-mounted in order to focus on the amount of two perichondral bones, the hyomandibular (Hm) around the hyosymplectic cartilage and the ceratohyal bone (Chb) around the ceratohyal cartilage. (This particular sample is also used for panels H and I.) Examples of Hm's scored as 0 (B), 1 (D), 2 (F), or 3 (H), and Chb's scored as 0 (C), 1 (E), 2 (G), or 3 (I). Some overlap of images in B-I occur, since the ceratohyal and hyosymplectic images are from the same sample. Scale bars: A=200µm; B-I=50µm. Abbreviations: A=anterior; AB/AR=Alcian blue/Alizarin red; Ch=ceratohyal cartilage; Chb=ceratohyal bone; Hm=hyomandibular bone; Hs=hyosymplectic cartilage; L=lateral.

Development • Supplementary information

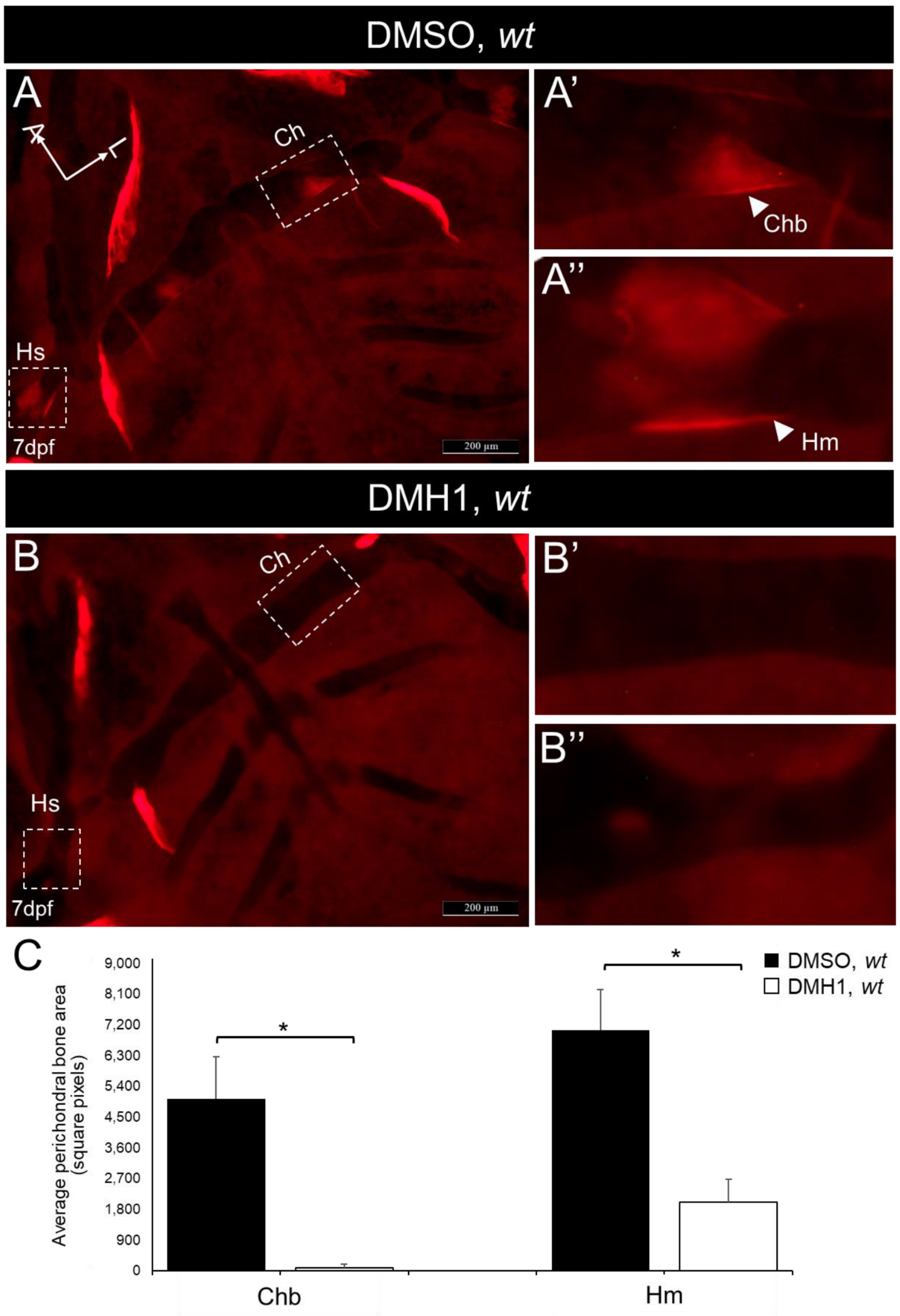

Fig. S3. DMH1 treatment blocks perichondral bone formation in wild-type cranial cartilage. (A-B'') Compared to DMSO-treated wild-type controls (A-A''), DMH1 treatment appeared to decrease perichondral bone in wild types at 7dpf (i.e., 3 days after treatment ended; B-B''). **(C)** Quantitation of Alizarin red fluorescent area of cartilages in 20 larvae for each experimental group confirmed a significant decrease in perichondral bone in DMH1-treated wild types (*, $p<0.05$). Scale bars: A,B=200µm. Abbreviations: A=anterior; Ch=ceratohyal cartilage; Chb=ceratohyal bone; Hm=hyomandibular bone; Hs=hyosymplectic cartilage; L=lateral.

Development • Supplementary information

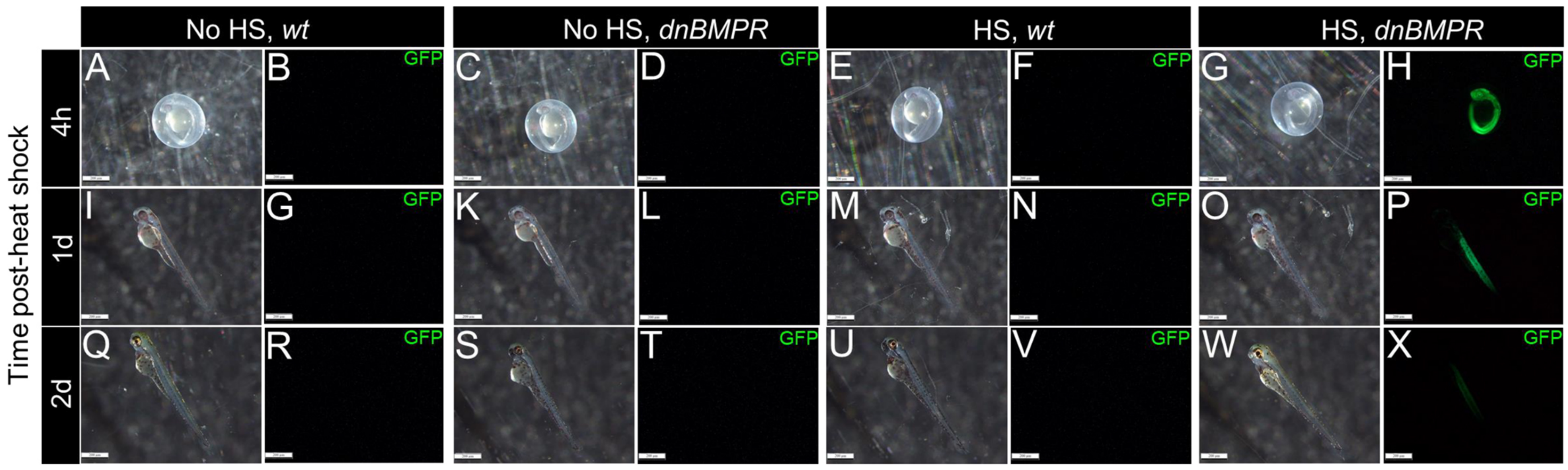

Fig. S4. *dnBMPR* transgene expression is inducible by heat shock. In contrast to no GFP detected in non-heat-shocked wild-type embryos (A,B,I,J,Q,R) non-heat-shocked transgenic embryos (C,D,K,L,S,T), or heat-shocked wild-type embryos (E,F,M,N,U,V), *dnBMPR* embryos that were heat-shocked at 1dpf exhibited GFP expression at 4 hours (G,H), 1 day (O,P), or 2 days (W,X) after heat shock. All zebrafish shown are from a single clutch. Scale bars: A-X=100µm. Abbreviations: d=day(s); h=hours; HS=heat-shocked.

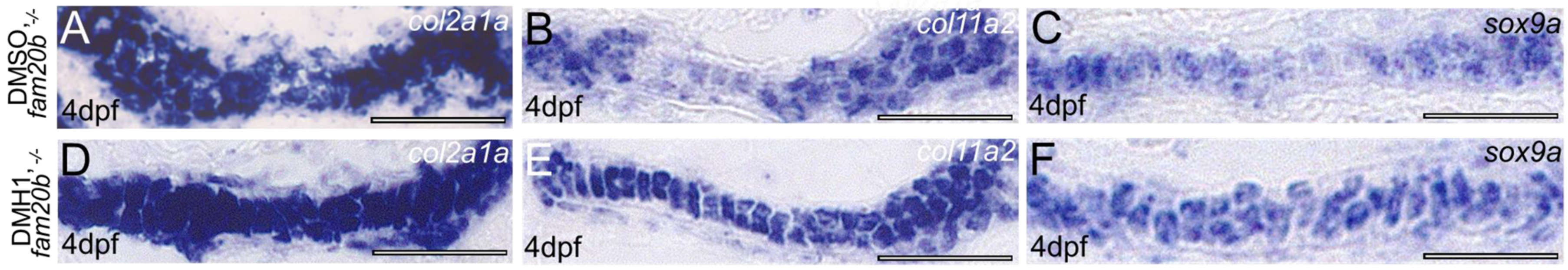

Fig. S5. DMH1 treatment rescues early chondrocyte maturation in *fam20b* mutants. (A-F) Compared to the downregulated expression of the genes *col2a1a* (A), *col11a2* (B) or *sox9a* (C) in DMSO-treated *fam20b*$^{-/-}$ mature chondrocytes, DMH1-treated *fam20b*$^{-/-}$ chondrocytes (D-F) did not downregulate these markers at 4dpf (i.e., after 48 hours of DMH1 treatment; these are representative images from at least 6 samples for each group). Scale bars: A-F=100µm.

Development • Supplementary information

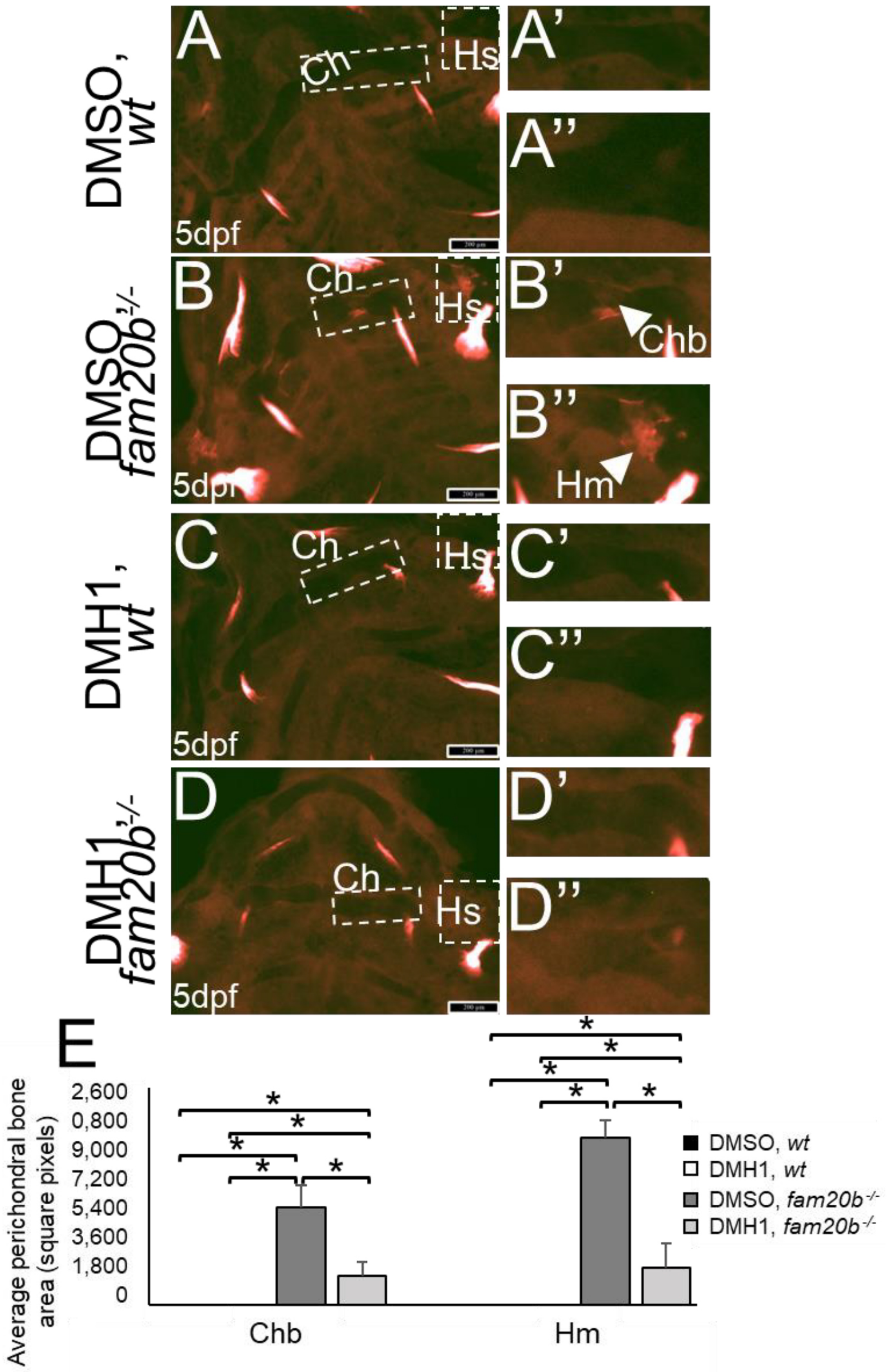

Fig. S6. DMH1 treatment rescues early perichondral bone in *fam20b* mutants. (A-B'') Neither DMSO-treated wild types (A-A'') nor DMH1-treated wild types (C-C'') demonstrated any perichondral bone at 5dpf (i.e., 1 day after treatment ended). Compared to DMSO-treated *fam20b* mutants (B-B''), DMH1 treatment appeared to decrease perichondral bone at 5dpf (i.e., 1 day after treatment ended; D-D''). **(E)** Quantitation of Alizarin red fluorescent area of cartilages in 20 larvae for each experimental group confirmed a significant decrease in perichondral bone in DMH1-treated *fam20b* mutants (*, $p<0.05$). Scale bars: A-D=200μm. Abbreviations: A=anterior; Ch=ceratohyal cartilage; Chb=ceratohyal bone; Hm=hyomandibular bone; Hs=hyosymplectic cartilage; L=lateral.

Development • Supplementary information

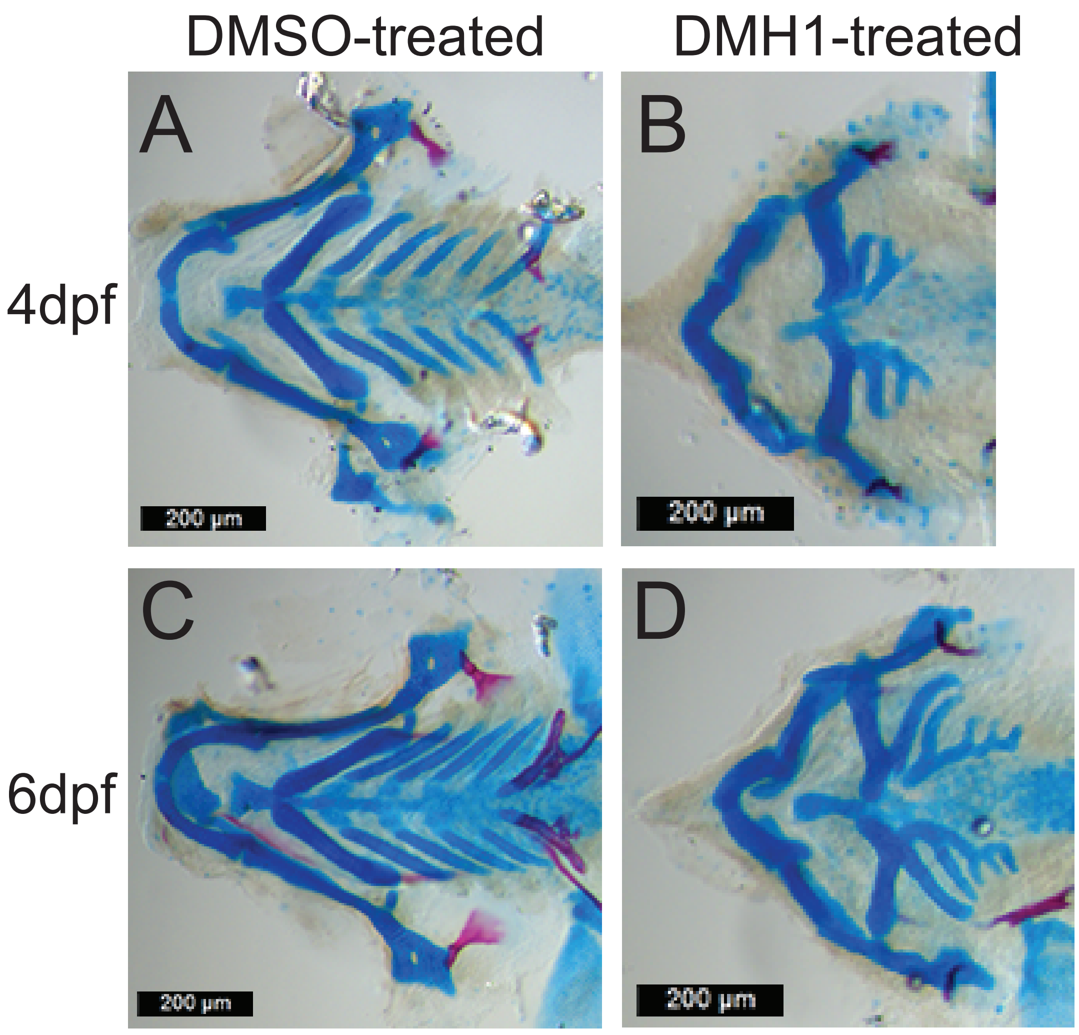

Fig. S7. DMH1 treatment at 1dpf causes morphological defects. Treatment with 10 µM DMH1 or an equivalent volume of DMSO began at 1dpf and was maintained for 48h. Larvae were fixed, stained with Alcian blue/Alizarin red, and pharyngeal skeletons were dissected. Compared to DMSO-treated embryos at 4dpf (A) and 6dpf (C), DMH1-treated embryos at 4dpf (B) and 6dpf (D) displayed delayed development and fusions of multiple skeletal elements.

Development • Supplementary information

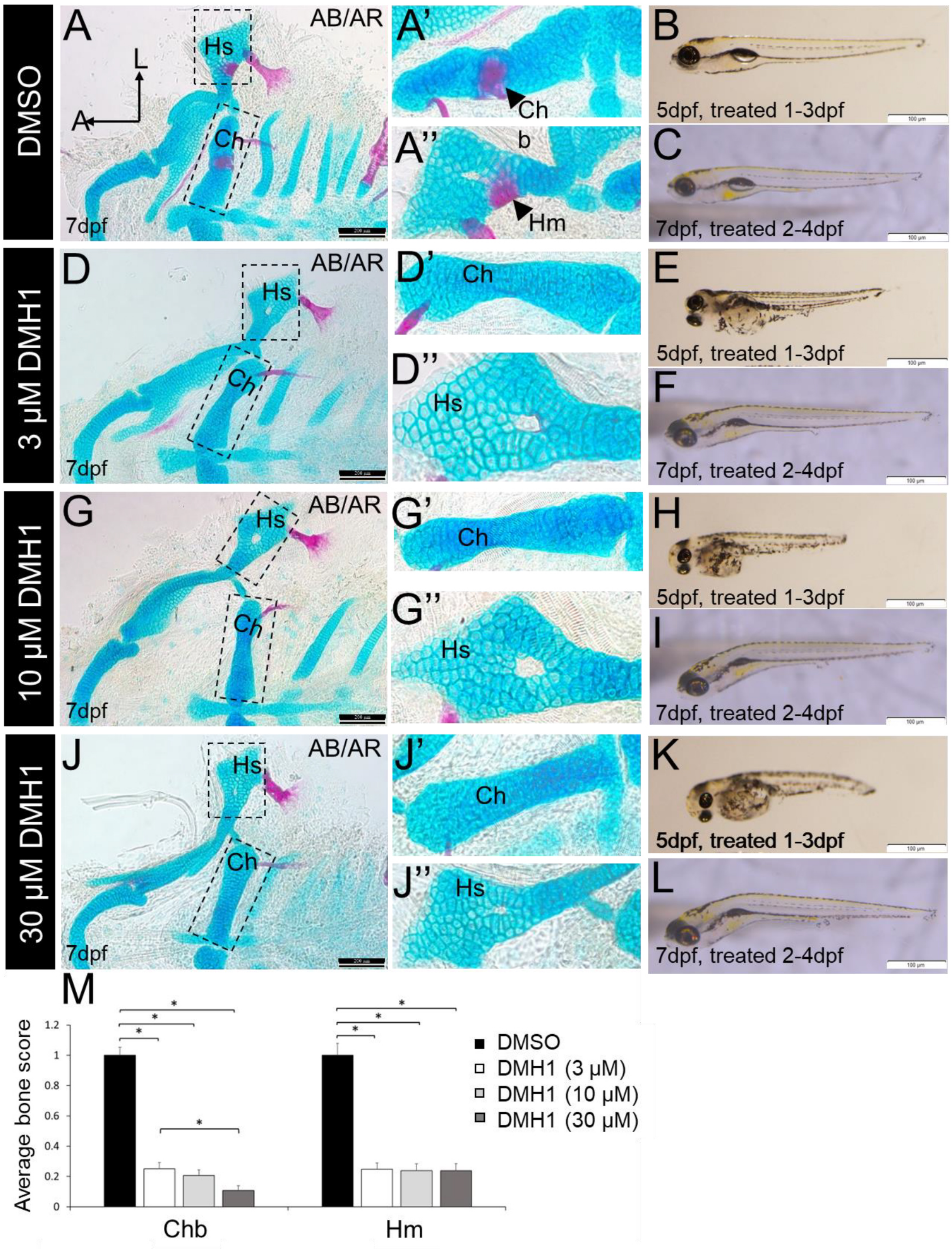

Fig. S8. DMH1 treatment decreases perichondral bone formation. Treatment with 3 different concentrations of DMH1 (3µM, 10µM, 30µM) from 48hpf to 96hpf decreased perichondral bone in wild-type zebrafish embryos at 7dpf. (5 clutches of 80 embryos each were scored) (*, $p<0.05$). Scale bars: A,D,G,J=200µm; B,C,E,F,H,I,K,L=100µm. Abbreviations: A=anterior; AB/AR=Alcian blue/Alizarin red; Ch=ceratohyal cartilage; Chb=ceratohyal bone; Hm=hyomandibular bone; Hs=hyosymplectic cartilage; L=lateral.

Development • Supplementary information

Table S1. Normalized counts for genes in the BMP signalling pathway from cranial cartilage of 6dpf wild-type zebrafish

Gene_Name	*Wild-type*	*Wild-type*	*Wild-type*	Average/*Wild-type*
Smad1	952	701	590	748
Smad5	141	63	42	82
Smad6a	25	45	31	34
Smad6b	41	323	113	159
Smad7	10	73	54	46
Smad9	29	37	10	25
Smad4 (3 of 6)	0	0	11	4
BMP1a	1574	1639	1737	1650
BMP2a	9	9	6	8
BMP2b	26	57	157	80
BMP2k	12	49	6	22
BMP4	4	1	1	2
BMP6	161	126	82	123
BMP7b	7	12	1	7
BMP8a	219	190	23	144
BMPR1aa	101	61	76	79
BMPR1ab	331	212	243	262
BMPR1ba	281	379	251	304
BMPR1bb	24	22	137	61
BMPR2a	115	219	98	144
BMPR2b	100	226	72	133
ihha	6364	3685	11779	7276
ihhb	57	32	261	117
id1	3283	2271	3273	2942
id2a	967	1781	859	1202
id2b	57	39	35	44
id3	1473	1169	1391	1344
id4	113	73	125	104
Smurf1	231	360	112	234
Smurf2	424	174	270	289
gadd45ab	577	214	802	531
gadd45ba	2205	10521	7079	6602
gadd45bb	172	954	796	641
gadd45g	388	648	834	623
gata1a	5	5	24	11
gata1b	11	16	4	10
gata2a	88	36	24	49
gata3	38	391	234	221
dkk3b	237	119	100	152
sost	10	1	10	7
