# Supplementary material for: Proteoglycan inhibition of canonical BMP-dependent cartilage maturation delays endochondral ossification
Source: Development. 2024 Jan 12;151(2):dev201716. doi: 10.1242/dev.201716 (PMC10820745; doi:10.1242/dev.201716)
Supplement: Supplementary file 1 [file develop-151-201716_review_history.pdf]

Proteoglycan inhibition of canonical BMP-dependent cartilage maturation delays endochondral ossification

Elham Koosha, Connor T. A. Brenna, Amir M. Ashique, Niteesh Jain, Katie Ovens, Toshiyasu Koike, Hiroshi Kitagawa and B. Frank Eames
DOI: 10.1242/dev.201716

Editor: Patrick Tam

Review timeline

Original submission:	17 February 2023
Editorial decision:	27 March 2023
First revision received:	24 October 2023
Editorial decision:	20 November 2023
Second revision received:	27 November 2023
Accepted:	28 November 2023

Original submission

First decision letter

MS ID#: DEVELOP/2023/201716

MS TITLE: Proteoglycan inhibition of canonical BMP-dependent cartilage maturation delays endochondral ossification

AUTHORS: Elham Koosha, Connor T. A. Brenna, Niteesh Jain, Toshiyasu Koike, Hiroshi Kitagawa and B. Frank Eames

I have now received all the referees' reports on the above manuscript, and have reached a decision. The referees' comments are appended below, or you can access them online: please go to BenchPress and click on the 'Manuscripts with Decisions' queue in the Author Area.

As you will see, the referees express considerable interest in your work, but have some significant criticisms and recommend a substantial revision of your manuscript before we can consider publication. If you are able to revise the manuscript along the lines suggested, which may involve further experiments, I will be happy receive a revised version of the manuscript. Your revised paper will be re-reviewed by one or more of the original referees, and acceptance of your manuscript will depend on your addressing satisfactorily the reviewers' major concerns. Please also note that Development will normally permit only one round of major revision. If it would be helpful, you are welcome to contact us to discuss your revision in greater detail. Please send us a point-by-point response indicating your plans for addressing the referee’s comments, and we will look over this and provide further guidance.

Please attend to all of the reviewers' comments and ensure that you clearly highlight all changes made in the revised manuscript. Please avoid using 'Tracked changes' in Word files as these are lost in PDF conversion. I should be grateful if you would also provide a point-by-point response detailing how you have dealt with the points raised by the reviewers in the 'Response to Reviewers' box. If you do not agree with any of their criticisms or suggestions please explain clearly why this is so.

Reviewer 1

© 2024. Published by The Company of Biologists under the terms of the Creative Commons Attribution License (https://creativecommons.org/licenses/by/4.0/).

Advance summary and potential significance to field

This paper examines the relationship between proteoglycan synthesis and BMP signaling in regulation of chondrocyte hypertrophy and bone formation in the perichondrium surrounding the cartilage. While a good deal is already known about the role of proteoglycans and BMP signaling in regulating endochondral bone development, this study makes good use of a fam20b kinase mutant that prevents nearly all proteoglycan synthesis in zebrafish. Two major findings are that BMP signaling is upregulated when proteoglycans are reduced in fam20b mutants, and that blocking BMP can rescue the endochondral bone defects of proteoglycan-deficient mutants.

Comments for the author

The experiments are well performed, controlled, and quantified. That said, the study feels somewhat limited in impact and scope. Figure 1 is validation of reduced kinase activity in two pre-existing fam20b mutants, Figures 2-4 show upregulated Bmp signaling in mutants and could be combined, Figure 5 shows that a known BMP inhibitor blocks endochondral ossification as expected, Figures 6-9 show that a published dnBmpr1 line blocks BMP signaling and endochondral ossification as expected and could be combined. Figures 10 and 11 are the most impactful as they show that blocking BMP signaling can restore normal endochondral bone development to a proteoglycan-deficient mutant. In general, this seems like a well performed and conclusive short article that moderately advances our understanding of endochondral bone development.

1. Last sentence of Abstract and last paragraph of Discussion concerning arthritis should be deleted as they are overly speculative given this paper deals with endochondral ossification and not joint development per se.

2. Lines 187-188 are misleading as it is stated that embryos are treated for 48 hours starting at 48hpf, but then defects at 72hpf are described later in paragraph (assuming 24 hour treatment?). More precise language should be used as to the different treatment/assay times, and the lengths of heat-shocks for each experiment should be explicitly stated in the figures themselves to make it easier to follow. For all figures, time-window for DMH1 treatment and dnBmpr heat-shock should be clearly defined in the legend (and where appropriate in the figures themselves).

3. Figure 1 would benefit from a diagram showing the known role of Fam20b in PG synthesis, as well as the kinase assay being performed.

4. Fig 2A,E - it is difficult to appreciate what are the cartilage PGs being secreted only at 72hpf - is this the reddish stain around the chondrocytes? If so it is very subtle and hard to recognize at the current magnification.

5. Should “Hmb” be used for hyomandibular bone since “Chb” used for ceratohyal bone?

Reviewer 2

Advance summary and potential significance to field

The manuscript “Proteoglycan inhibition of canonical BMP-dependent cartilage maturation delays endochondral ossification” investigates the role of proteoglycan inhibition of BMP signaling in cartilage maturation. The authors use mainly immunostaining and in situ probes to characterize the timing of BMP signaling in zebrafish embryos. Analysis of cartilage maturation in zebrafish fam20b mutants which have reduced proteoglycan biosynthesis and premature cartilage maturation show that BMP signaling is prematurely initiated in chondrocytes. The authors then take advantage of a BMP signaling inhibitor and an inducible dominant negative BMPR transgene line to reduce the premature onset of BMP signaling and bone formation in the fam20b mutants.

The experiments are well executed and coherent. The main weakness of the study is the relatively low tech methods used with its focus on a couple of markers important for the authors hypothesis. Proteoglycan functions are extremely broad and it is quite likely that signaling by FgF, Wnt, Hh and

© 2024. Published by The Company of Biologists under the terms of the Creative Commons Attribution License (https://creativecommons.org/licenses/by/4.0/).

other paracrine factors are also affected as well as other effects on chondrocytes by the changed composition of extracellular matrix. The study aims to single out BMP signaling as a major regulator of timing of bone formation, and does it somewhat successfully but an extended analysis of cartilage tissues in the rescue experiments would likely present a more complete picture.

Comments for the author

Main Points.

1. I'm do not find the title ideal. It is not incorrect but it somehow gives the impression of a discovery of an instructive mechanism for proteoglycans in inhibiting BMP signaling to delay endochondral ossification - I don't think it is what this paper has discovered. The proteoglycan presence in cartilage is massive and multifunctional and nothing in this study suggest anything but proteoglycans to be a stable permissive condition for BMP-dependent cartilage maturation. A title in the line of "Impaired proteoglycan biosynthesis induce premature canonical BMP-dependent cartilage maturation and endochondral ossification" or "Proteoglycans are necessary for timing of cartilage maturation" should be considered. But I'm open for arguments.
2. To me the strongest evidence in the study is the rescue of timing of cartilage maturation genes and bone formation. Yet the nature of the rescue phenotype is not described in much detail apart from two in situs and alizarin staining. It is highly likely that a large number of signaling factors are affected by the strong reduction of PGs fam20b mutants.
- Is the rescue limited to just the few aspects of restored markers and the timing of initiation of bone formation?
- Or is the BMP inhibition by transgenics and inhibitor effectively restoring the general timing of the maturation, singling out BMP as the key regulator of this process?

Is the rescue, either by inhibitor or the dominant negative transgene, able to restore some morphological aspect of WT cartilage elements (for example number of cells, cell shape, cartilage element size or shape or aspects of chondrocyte ultrastructure) that is lost in fam20b mutants? Or is it possible to provide a broader analysis of the transcriptome changes in mutants and rescued mutants vs wild types? I would like to see some substantial expansion on one or several of these aspects.

3. The scoring system for perichondral bone feels arbitrary risking unintentional subjective errors in quantification (supplemental figure 2). It should be replaced by a robust objective measure (e.g. number/proportion of alizarin stained cells in element x or alizarin stained x% of total surface area of flat-mounted element.)

Minor points
1. I don't understand this sentence at row 78: " although Smad deletion in limb cartilage was slightly delayed technically [27]."
2. Are 5dpf alizarin staining completely blank in ceratohyal bone and hyosymplectic cartilage? I don't see that control but maybe I missed this.

<u>Reviewer 3</u>

Advance summary and potential significance to field

In this clearly-written manuscript by Koosha and colleagues, the authors use zebrafish mutants paired with drug treatments and dominant-negative transgenes to test the hypothesis that proteoglycans affect bone formation by modulating BMP signaling to chondrocytes. Understanding this process is important because changes in proteoglycans may underlie bone diseases like osteoarthritis. Thus the paper is significant for the field. The advance made in this paper and potential significance to the field are appropriate for the Development readership. However, I suggest a few additions that would strengthen the model and provide a deeper mechanistic understanding. I provide three major issues that should be resolved, and many minor points that also need to be addressed.
In summary, this is a good paper with strong data addressing an interesting topic, but it needs only a few revisions to propel it into a top-tier journal like Development.

Comments for the author

© 2024. Published by The Company of Biologists under the terms of the Creative Commons Attribution License (https://creativecommons.org/licenses/by/4.0/).

Issues that must first be addressed:

Major Issue 1: In this system (ceratohyal ossification), which cells are BMP signal-sending and which are BMP signal-receiving? While there are some analyses of which cells are BMP signal receiving (chondrocytes not perichondral cells), overall the details of the signaling in this system are not sufficiently addressed. A major missing piece in the model is uncovering where the BMP ligand(s) are coming from. Similarly, which BMPs and BMPRs are involved? Some potential approaches that may uncover which cells are ligand (sending) and which are receptor (receiving) include in situ hybridization or antibody stains for BMPs and BMPRs, transplantation or transgenic mosaic analyses. Mining existing datasets like those in “Lifelong single-cell profiling of cranial neural crest diversification in zebrafish” by Fabian et al 2022 could provide insight into which cells are expressing which ligands and receptors in the system.

Major issue 2: What do the Smad subcellular localization changes mean for the model? Addressing the canonical (pSmad1/5/9) vs noncanonical (p-p38) is a strength. However, the relevance of the nuclear vs non-nuclear pSmad1/5/9 is not clear. This concept needs to be clearly set up in the introduction so it can be put into context in the results. These findings are intriguing, but what they mean for the model is not fully fleshed out. In this same vein, the experiments in supplementary Fig. 1 are critical controls that strengthen the argument, but the images are low resolution, so it is impossible to discern if the signal is nuclear or non-nuclear. This distinction is claimed to be important in the cartilage (although I’m not clear why) so it would be nice to know which is being labeled in the trunk. Is the idea that pSmad is restricted to the cytoplasm in the absence of BMP in this system? Maybe cytoplasm vs nucleus is two stages of early vs late BMPR activation? This needs to be clarified.
Further, the drug and the DN construct are transient treatments and the authors make the point that the effect is likely short term. Do the fam mutant phenotypes revert back later? Or does BMP inhibition have a lasting effect on the phenotype even after the drug or transgene have worn off?

Major issue 3: is the phenotype in the fam mutants due to just changes in BMP?
In figure 11 the wild type siblings need to be included so the reader can observe the difference between wild-type condition, vs mutant condition, vs rescue (BMP inhibition) condition. For example, is the rescue condition similar to wild type? Or is there still ectopic BMP activation, less than wild type?
Same thing with the skeletal preps. Does the BMP inhibition bring the amount of chondral bone back to WT levels? Below WT levels? This cannot be discerned from the data since only data on fam20b -/- (and not the wild type) are presented.
This is not just cosmetic, comparing these conditions may inform if other molecules are involved. For example, if inhibiting BMP produces a full rescue that would indicate that ONLY the increase in BMP signaling produces the bone phenotype. In contrast, a partial rescue would indicate that there are other factors affecting bone development in the proteoglycan mutants besides just BMP.

Additional minor comments:
I applaud the biochemical analyses of the previously published zebrafish alleles, this is a nice advance. But the data seem preliminary and underdeveloped. The reader needs more information on this assay so they can understand the conclusions and how they relate to the big picture story. Also the generation of cDNA encoding truncated forms of Fam20b that are secreted seems questionable. The authors dropped the first 29 N term amino acids, but is the same difference in activity seen with the wild type vs the two mutants when full length proteins are analyzed? At a minimum there needs to be some controls in the current assay that the amount of kinase is consistent across conditions and the only difference is in actual activity. There do not appear to be any controls in this experiment. Transfection amount needs to be normalized, as well as the amount of kinase present in different conditions etc. The number of replicates was not easily found.

Controversy regarding role of BMP: “To clarify discrepancies in published reports” It was not initially clear to me that there is controversy regarding the role of PG and BMP at this point in the paper. This should be more clearly set up or better yet explicitly stated in the introduction as motivation for the study.

© 2024. Published by The Company of Biologists under the terms of the Creative Commons Attribution License (https://creativecommons.org/licenses/by/4.0/).

In Fig 2 rather than citing “fam20b-/- chondrocytes also differentiate according to this timing [5].” It would be more rigorous to include mutant siblings alongside the wild types for the safranin experiment. That way the reader can directly compare the precise timing differences between wild types and mutants.
Timing is such a big part of this work, these data are curiously omitted. This addition is important for context for the rest of the figure too.

“At this time (48 hpf), mesenchymal condensation of specified chondrocytes had already occurred (Fig. 2A)”, this statement isn’t supported by the data. These sections do not illustrate chondrocyte condensation or differentiation. Of note it is a bit confusing because the authors previously used these data to illustrate that cartilage PGs are not being produced at this stage. I realize that they are arguing that the earliest stages of chondrocyte differentiation are occurring at the 48 hpf, as opposed to actively secreting PGs, but this one section without any molecular markers is not sufficient to demonstrate that there are mesenchymal cells becoming chondrocytes at this stage. Some kind of molecular marker of identity is required

“Molecular and histological markers demonstrated a delay in endochondral ossification after DMH1 treatment” Is this supposed to be a reference from previous work? This hasn’t been shown in this manuscript yet.

That the drug and DNBMP treatments rescue fam mutants is convincing.

“The amount of perichondral bone, such as seen in either the ceratohyal or hyomandibular, appeared reduced in the craniofacial skeleton of DMH1-treated larvae at 7dpf, compared to DMSO-treated 203 controls (Fig. 5J-K’’)”

I would point out the opercle and branchiostegal ray dermal bones are unaffected. This is an excellent internal control demonstrating tissue specific defects and not some global change. On this note, can you perform some quantification of dermal bone mineralization score that would be unchanged to drive this point home?

The heat shock induction experiment in supplemental fig. 3 is good, but an important control is missing, the non heat shock transgene to show the amount of leakiness from this transgene without heat shock. Especially because this condition is presented in figure 6 it is important to know if there are basal levels of dnBMPR in these conditions, I suspect there is since heat shock driven transgenes can sometimes be expressed in the absence of heat shock. In fact, in fig 6B there may be reduced p-Smad staining reflecting a constitutive, low level, blockade in this genotype. Because timing is so important to this model if the treatment really is acute or sustained is an important point.

All zebrafish images should be oriented per convention with anterior to the left. This makes the images and the figures easier for the reader to interpret.

A couple of sentences early in the introduction describing exactly what proteoglycans are would be helpful for non-expert, general, readers of Development. Core proteins, repeating glycosaminoglycans etc.

First revision

Author response to reviewers' comments

Response to Reviewers

Koosha et al. DEVELOP/2023/201716

Reviewer 1 Advance Summary and Potential Significance to Field:

© 2024. Published by The Company of Biologists under the terms of the Creative Commons Attribution License (https://creativecommons.org/licenses/by/4.0/).

This paper examines the relationship between proteoglycan synthesis and BMP signaling in regulation of chondrocyte hypertrophy and bone formation in the perichondrium surrounding the cartilage. While a good deal is already known about the role of proteoglycans and BMP signaling in regulating endochondral bone development, this study makes good use of a fam20b kinase mutant that prevents nearly all proteoglycan synthesis in zebrafish. Two major findings are that BMP signaling is upregulated when proteoglycans are reduced in fam20b mutants, and that blocking BMP can rescue the endochondral bone defects of proteoglycan- deficient mutants.

Reviewer 1 Comments for the Author: The experiments are well performed, controlled, and quantified. That said, the study feels somewhat limited in impact and scope. Figure 1 is validation of reduced kinase activity in two pre-existing fam20b mutants, Figures 2-4 show upregulated Bmp signaling in mutants and could be combined, Figure 5 shows that a known BMP inhibitor blocks endochondral ossification as expected, Figures 6-9 show that a published dnBmpr1 line blocks BMP signaling and endochondral ossification as expected and could be combined. Figures 10 and 11 are the most impactful as they show that blocking BMP signaling can restore normal endochondral bone development to a proteoglycan-deficient mutant. In general, this seems like a well performed and conclusive short article that moderately advances our understanding of endochondral bone development.

We thank the reviewer for their careful consideration and nice words about our paper.

1. Last sentence of Abstract and last paragraph of Discussion concerning arthritis should be deleted as they are overly speculative given this paper deals with endochondral ossification and not joint development per se.

Many researchers have argued that chondrocyte maturation shares the same developmental pathway during articular cartilage development as during primary ossification along the mid-diaphysis. Reviewer #3 here supported the relevance of this study to osteoarthritis. Accordingly, we hope that this reviewer accepts our motivation that insights, such as ours here, exposing how PGs and Bmp's interact to regulate chondrocyte maturation are indeed relevant to developing therapeutics for the aberrant maturation seen in OA. We prefer to leave those sentences in our manuscript.

2. Lines 187-188 are misleading as it is stated that embryos are treated for 48 hours starting at 48hpf, but then defects at 72hpf are described later in paragraph (assuming 24 hour treatment?). More precise language should be used as to the different treatment/assay times, and the lengths of heat-shocks for each experiment should be explicitly stated in the figures themselves to make it easier to follow. For all figures, time-window for DMH1 treatment and dnBmpr heat-shock should be clearly defined in the legend (and where appropriate in the figures themselves).

We agree that this comment could help clarify much to the reader, so in response, we added clarification of treatment times throughout the results text and in figure legends.

3. Figure 1 would benefit from a diagram showing the known role of Fam20b in PG synthesis, as well as the kinase assay being performed.

In response, we have clarified the Fam20b biochemical function as requested schematically in the figure (Figure 1A) and described better in the Methods (lines 482- 493), Results (lines 164-166), and Figure legend (lines 775-791) the actual assay being performed.

4. Fig 2A,E - it is difficult to appreciate what are the cartilage PGs being secreted only at 72hpf - is this the reddish stain around the chondrocytes? If so, it is very subtle and hard to recognize at the current magnification.

We are sorry, but indeed, the subtle reddish stain around the chondrocytes is exactly what reflects cartilage PG secretion. In response to this request, we included a high- mag cropped image of the ceratohyal cartilage to better show the result (new Figure 2G'), also writing a better description of the result in the Results (lines 189-193) and Figure legend (lines 797-800).

© 2024. Published by The Company of Biologists under the terms of the Creative Commons Attribution License (https://creativecommons.org/licenses/by/4.0/).

5. Should “Hmb” be used for hyomandibular bone since “Chb” used for ceratohyal bone?

Sorry for any confusion in these terms, but there is a good reason for us doing this, following precedent in the FishFace Atlas (https://www.facebase.org/resources/zebrafish/fishface/home/). The cartilage element in the ventral second arch is called the ceratohyal, while the bone in the perichondrium of that element is also called the ceratohyal. To avoid confusion about which tissue we are focussed on, we inserted “bone” after ceratohyal to clarify that we mean the perichondral bone in the ceratohyal cartilage. Regarding the dorsal second arch element, the cartilage is called the hyosymplectic, while its perichondral bone is called the hyomandibular. Therefore, no extra word is needed in this case to clarify the tissue of interest.

Reviewer 2 Advance Summary and Potential Significance to Field:

The manuscript “Proteoglycan inhibition of canonical BMP-dependent cartilage maturation delays endochondral ossification” investigates the role of proteoglycan inhibition of BMP signaling in cartilage maturation. The authors use mainly immunostaining and in situ probes to characterize the timing of BMP signaling in zebrafish embryos. Analysis of cartilage maturation in zebrafish fam20b mutants, which have reduced proteoglycan biosynthesis and premature cartilage maturation, show that BMP signaling is prematurely initiated in chondrocytes. The authors then take advantage of a BMP signaling inhibitor and an inducible dominant negative BMPR transgene line to reduce the premature onset of BMP signaling and bone formation in the fam20b mutants.

The experiments are well executed and coherent. The main weakness of the study is the relatively low tech methods used with its focus on a couple of markers important for the authors hypothesis. Proteoglycan functions are extremely broad and it is quite likely that signaling by FgF, Wnt, Hh and other paracrine factors are also affected as well as other effects on chondrocytes by the changed composition of extracellular matrix. The study aims to single out BMP signaling as a major regulator of timing of bone formation, and does it somewhat successfully, but an extended analysis of cartilage tissues in the rescue experiments would likely present a more complete picture.

We thank the reviewer for nice comments on our study, and we hope that our additional experiments in this revision satisfy their requests.

Reviewer 2 Comments for the Author:

Main Points.

1. I’m do not find the title ideal. It is not incorrect but it somehow gives the impression of a discovery of an instructive mechanism for proteoglycans in inhibiting BMP signaling to delay endochondral ossification - I don’t think it is what this paper has discovered. The proteoglycan presence in cartilage is massive and multifunctional and nothing in this study suggest anything but proteoglycans to be a stable permissive condition for BMP- dependent cartilage maturation. A title in the line of “Impaired proteoglycan biosynthesis induce premature canonical BMP-dependent cartilage maturation and endochondral ossification” or “Proteoglycans are necessary for timing of cartilage maturation” should be considered. But I'm open for arguments.

Since the reviewer agrees that the current title is factually correct, we prefer to keep that title, especially since it adds a PG-growth factor mechanism to our 2011 *fam20b* paper in *PLoS Genetics*, whose title summarized that cartilage PGs alter the timing of endochondral ossification by inhibiting chondrocyte maturation.

But thanks so much for bringing up this very interesting conceptual discussion regarding instructive vs permissive interactions, my modern take of which I actually teach students in my undergrad course in Developmental Biology. To clarify for readers through what kind of interactions PGs regulate developmental timing, we responded by adding the reviewer’s point to the paper’s discussion (lines 372-381):

© 2024. Published by The Company of Biologists under the terms of the Creative Commons Attribution License (https://creativecommons.org/licenses/by/4.0/).

"More broadly, the role of PGs in skeletal development traditionally would be described as a permissive interaction (Gilbert and Barresi, 2000), merely providing an environment that permits instructive cues (e.g., growth factors) to reach their target cells. However, our data argue that at least some biological roles of extracellular matrix should no longer be considered permissive interactions. As shown here and discussed below, the levels of PGs can affect the timing of skeletal development in an instructive fashion. In wild types, the levels of cartilage PGs have been calibrated through evolution to regulate growth factor signalling and achieve "normal" timing of skeletal development, whereas in *fam20b* mutants, the levels of cartilage PGs are decreased, and skeletal development is accelerated."

2. To me the strongest evidence in the study is the rescue of timing of cartilage maturation genes and bone formation. Yet the nature of the rescue phenotype is not described in much detail apart from two in situs and alizarin staining. It is highly likely that a large number of signaling factors are affected by the strong reduction of PGs fam20b mutants. -Is the rescue limited to just the few aspects of restored markers and the timing of initiation of bone formation? -Or is the BMP inhibition by transgenics and inhibitor effectively restoring the general timing of the maturation, singling out BMP as the key regulator of this process? Is the rescue, either by inhibitor or the dominant negative transgene, able to restore some morphological aspect of WT cartilage elements (for example number of cells, cell shape, cartilage element size or shape or aspects of chondrocyte ultrastructure) that is lost in fam20b mutants? Or is it possible to provide a broader analysis of the transcriptome changes in mutants and rescued mutants vs wild types? I would like to see some substantial expansion on one or several of these aspects.

These are great points, and indeed, we are working on another study that evaluates additional signalling pathways in *fam20b* mutant cartilage development. So, we hope the reviewer can be satisfied with the additional experiments we have done in response to these points. We expanded *in situ* analyses of maturation with three additional markers that are normally down-regulated during maturation: *col2a1a*, *col11a2*, and *sox9a*. Compared to DMSO-treated *fam20b* mutants, who downregulate these markers in mature cartilage at 4dpf, DMH1-treated *fam20b* mutants do not downregulate these markers (lines 317-321; new Supplemental Figure 5). These data on downregulated genes show the opposite effect from our analyses of the upregulated genes *ihha* and *col10a1a*, providing strong further support for our conclusion. In addition, we expanded upon the nature of the rescue phenotype by evaluating the degree of perichondral bone rescue of our DMH1 treatments of *fam20b* mutants at a much later timepoint (10dpf).
Interestingly, we found that the amount of perichondral bone was similar to wild-type controls (lines 328-334; new Figure 13). We also added an emphasis on this result in the Discussion, since it might suggest that early disease treatment may have long- lasting effects (lines 406-408).

3. The scoring system for perichondral bone feels arbitrary risking unintentional subjective errors in quantification (supplemental figure 2). It should be replaced by a robust objective measure (e.g. number/proportion of alizarin stained cells in element x or alizarin stained x% of total surface area of flat-mounted element.)

We agree with this rigorous point. In response, we have added quantitative measures of the numbers of pixels of perichondral bone from Alizarin red fluorescent images, which confirmed the findings using the previous scoring system (lines 260-263; 322- 325; new Supplemental Figures 6&7). We also employed this fluorescent image quantitation in our new 10dpf "rescue" experiments (lines 328-334; new Figure 13).

Minor points

1. I don't understand this sentence at row 78: " although Smad deletion in limb cartilage was slightly delayed technically [27]."

This phrase was removed.

2. Are 5dpf alizarin staining completely blank in ceratohyal bone and hyosymplectic cartilage? I don't see that control but maybe I missed this.

Yes, that's right; wild-type 5dpf larvae do not typically stain for Alizarin red in the ceratohyal bone

© 2024. Published by The Company of Biologists under the terms of the Creative Commons Attribution License (https://creativecommons.org/licenses/by/4.0/).

or hyomandibular in our facility. To respond directly to this point, we included this control in our fluorescent bone analyses (new Supplemental Figure 7).

Reviewer 3 Advance Summary and Potential Significance to Field:

In this clearly-written manuscript by Koosha and colleagues, the authors use zebrafish mutants paired with drug treatments and dominant-negative transgenes to test the hypothesis that proteoglycans affect bone formation by modulating BMP signaling to chondrocytes. Understanding this process is important because changes in proteoglycans may underlie bone diseases like osteoarthritis. Thus, the paper is significant for the field. The advance made in this paper and potential significance to the field are appropriate for the Development readership. However, I suggest a few additions that would strengthen the model and provide a deeper mechanistic understanding. I provide three major issues that should be resolved, and many minor points that also need to be addressed. In summary, this is a good paper with strong data addressing an interesting topic, but it needs only a few revisions to propel it into a top-tier journal like Development.

We thank the reviewer for appreciating the significance of our paper, especially supporting the paper's relevance to osteoarthritis, and we hope that the many experiments that we added in this revision have resolved all of the points raised below.

Reviewer 3 Comments for the Author:

Issues that must first be addressed:

Major Issue 1: In this system (ceratohyal ossification), which cells are BMP signal- sending and which are BMP signal-receiving? While there are some analyses of which cells are BMP signal receiving (chondrocytes not perichondral cells), overall the details of the signaling in this system are not sufficiently addressed. A major missing piece in the model is uncovering where the BMP ligand(s) are coming from. Similarly, which BMPs and BMPRs are involved? Some potential approaches that may uncover which cells are ligand (sending) and which are receptor (receiving) include in situ hybridization or antibody stains for BMPs and BMPRs, transplantation or transgenic mosaic analyses. Mining existing datasets like those in "Lifelong single-cell profiling of cranial neural crest diversification in zebrafish" by Fabian et al 2022 could provide insight into which cells are expressing which ligands and receptors in the system.

To respond to this important point, we generated a novel RNAseq laser capture microdissection dataset from cranial cartilage in the 6dpf zebrafish (new Supplemental Table 1). These data demonstrated expression of several components of the BMP signalling pathway in developing cranial chondrocytes, including ligands, receptors, and intracellular cascade members (lines 176-183).

Major issue 2: What do the Smad subcellular localization changes mean for the model? Addressing the canonical (pSmad1/5/9) vs noncanonical (p-p38) is a strength. However, the relevance of the nuclear vs non-nuclear pSmad1/5/9 is not clear. This concept needs to be clearly set up in the introduction so it can be put into context in the results. These findings are intriguing, but what they mean for the model is not fully fleshed out. In this same vein, the experiments in supplementary Fig. 1 are critical controls that strengthen the argument, but the images are low resolution, so it is impossible to discern if the signal is nuclear or non-nuclear. This distinction is claimed to be important in the cartilage (although I'm not clear why) so it would be nice to know which is being labeled in the trunk. Is the idea that pSmad is restricted to the cytoplasm in the absence of BMP in this system? Maybe cytoplasm vs nucleus is two stages of early vs late BMPR activation? This needs to be clarified.

We thank the reviewer tremendously for pointing out our omission of the significance of nuclear translocation of phosphorylated Smad1/5/9 or p38, especially since it was directly addressed by our image analyses. In response to this comment, we have added the following text to the Introduction: "Upon phosphorylation by activated BMP receptors, Smad1/5/9 or p38 move from the cytoplasm to the nucleus, where they act as transcription factors to regulate gene expression [23]." (lines 86-88). The images in Supplemental Figure 1 were merely to demonstrate that the

© 2024. Published by The Company of Biologists under the terms of the Creative Commons Attribution License (https://creativecommons.org/licenses/by/4.0/).

inhibition of BMP signalling was working at the early timepoint of 48hpf, even though we didn't see any effects on chondrocytes at this time, so lower resolution was sufficient to accomplish this limited objective. But in response to further reviewer comments, we added to the discussion about the potential significance of the timing of sub-cellular transport of Smad1/5/9: "Interestingly, p-Smad1/5/9 levels appeared to first increase in the cytoplasm of chondrocytes at 72hpf, but not in the nucleus. Significant increases in p-Smad1/5/9 levels in the nucleus were not observed until slightly later, at 84hpf. These observations suggest that nuclear translocation of activated Smads is inhibited when decreased PGs allow more BMP signalling to occur in early stages of chondrocyte differentiation.
Perhaps Smad1/5/9 have specific inhibitors of nuclear import in this context, similar to what has been proposed regarding Smad2/3 linker phosphorylation or Imp7/8 inhibition [58, 59]" (lines 414-421).

Further, the drug and the DN construct are transient treatments and the authors make the point that the effect is likely short term. Do the fam mutant phenotypes revert back later? Or does BMP inhibition have a lasting effect on the phenotype even after the drug or transgene have worn off?

In response to this important comment, we performed additional experiments, using Alizarin red fluorescent images to quantitate levels of perichondral bone at 10dpf in *fam20b* mutants that were DMH1-treated from 2-4dpf (as previously analyzed at 5dpf in Figure 12). As shown in new Figure 13, and presented in the text (lines 328-334), the levels of perichondral bone actually were statistically indistinguishable from untreated wt at 10dpf, remarkably suggesting that early and transient DMH1 treatment somehow had a lasting effect on the phenotype even after the drug was removed. Therefore, we also added this to the Discussion (lines 406-408): "Furthermore, our data on *fam20b* mutants at 10dpf (6 days after DMH1 treatment ended) suggested that even a short treatment period early in a disease might cause lasting, clinically-relevant effects".

Major issue 3: is the phenotype in the fam mutants due to just changes in BMP? In figure 11 the wild type siblings need to be included so the reader can observe the difference between wild-type condition, vs mutant condition, vs rescue (BMP inhibition) condition. For example, is the rescue condition similar to wild type? Or is there still ectopic BMP activation, less than wild type? Same thing with the skeletal preps. Does the BMP inhibition bring the amount of chondral bone back to WT levels? Below WT levels? This cannot be discerned from the data since only data on fam20b -/- (and not the wild type) are presented. This is not just cosmetic, comparing these conditions may inform if other molecules are involved. For example, if inhibiting BMP produces a full rescue that would indicate that ONLY the increase in BMP signaling produces the bone phenotype. In contrast, a partial rescue would indicate that there are other factors affecting bone development in the proteoglycan mutants besides just BMP.

To respond directly to this point, we included the wt control at 5dpf in our fluorescent bone analyses (Supplemental Figure 7). Indeed, they do not have any perichondral bone at this timepoint, so DMH1 treatment could not produce any changes. While DMH1 treatment of *fam20b* mutants did not remove all bone at 5dpf (which was not similar to untreated wt at this timepoint, who have no bone), our new data at 10dpf (discussed in previous comment) showed that levels of bone in DMH1-treated *fam20b* mutants were statistically indistinguishable from untreated wild-types. While we are very happy to obtain this result in response to the reviewer, we are reluctant to interpret this as meaning that BMP signalling is the only pathway altered in *fam20b* mutants. In a future study, we aim to determine other pathways altered in *fam20b* mutants.

Additional minor comments:

I applaud the biochemical analyses of the previously published zebrafish alleles, this is a nice advance. But the data seem preliminary and underdeveloped. The reader needs more information on this assay so they can understand the conclusions and how they relate to the big picture story. Also, the generation of cDNA encoding truncated forms of Fam20b that are secreted seems questionable. The authors dropped the first 29 N term amino acids, but is the same difference in activity seen with the wild type vs the two mutants when full length proteins are analyzed? At a minimum there needs to be some controls in the current assay that the amount of kinase is consistent across conditions and the only difference is in actual activity. There do not appear to be any controls in this experiment. Transfection amount needs to be normalized, as well as the

© 2024. Published by The Company of Biologists under the terms of the Creative Commons Attribution License (https://creativecommons.org/licenses/by/4.0/).

amount of kinase present in different conditions etc. The number of replicates was not easily found.

We thank the reviewer for acknowledging our efforts to couch our biological findings in a biochemical context of the specific mutations utilized in our zebrafish models. In response to this comment, we have clarified the Fam20b biochemical function schematically in the figure (Figure 1A), included a new image demonstrating how protein levels were controlled in the experiment (Figure 1B), and described the assay, relevant controls, and sample sizes much better in the Methods (lines 482-493), Results (lines 164-166), and Figure legend (lines 775-791). Our collaborators are experts in the field in the biochemistry of proteoglycan synthesis, and they have published these exact techniques widely in strictly biochemical and more general journals, so they are broadly accepted as valid biochemical approaches. In response to the reviewer comment, we added more references for these published techniques (Koike et al, 2009, 2014, 2022).

Controversy regarding role of BMP: “To clarify discrepancies in published reports” It was not initially clear to me that there is controversy regarding the role of PG and BMP at this point in the paper. This should be more clearly set up or better yet explicitly stated in the introduction as motivation for the study.

We added emphasis to the conflicting reports in the Introduction, as requested, modifying the topic sentence “How PGs affect BMP-dependent cartilage maturation is unclear, since published data show both positive and negative regulation” (lines 123- 124).

In Fig 2 rather than citing “fam20b-/- chondrocytes also differentiate according to this timing [5].” It would be more rigorous to include mutant siblings alongside the wild types for the safranin experiment. That way the reader can directly compare the precise timing differences between wild types and mutants. Timing is such a big part of this work, these data are curiously omitted. This addition is important for context for the rest of the figure too. “At this time (48 hpf), mesenchymal condensation of specified chondrocytes had already occurred (Fig. 2A)”, this statement isn’t supported by the data. These sections do not illustrate chondrocyte condensation or differentiation. Of note, it is a bit confusing because the authors previously used these data to illustrate that cartilage PGs are not being produced at this stage. I realize that they are arguing that the earliest stages of chondrocyte differentiation are occurring at the 48 hpf, as opposed to actively secreting PGs, but this one section without any molecular markers is not sufficient to demonstrate that there are mesenchymal cells becoming chondrocytes at this stage. Some kind of molecular marker of identity is required

In response to this important point, we performed additional experiments demonstrating that both histological and molecular features of the early stages of chondrogenesis appear to follow similar developmental timing, making this into a new Figure 2. We added *fam20b* mutant controls to the Safranin O staining at the two timepoints (new Fig. 2B,B’,H,H’). In addition, we performed molecular analyses to support the assertion that developing ceratohyal cells of both wild types and *fam20b* mutants were undergoing mesenchymal condensation at 48hpf. Supporting the morphological changes to the ceratohyal condensation seen in the Safranin O histology, those cells expressed early cartilage markers, such as *col2a1a* and *sox9a*, at much higher levels than surrounding mesenchyme (new Fig. 2C-F, lines 187-189).

“Molecular and histological markers demonstrated a delay in endochondral ossification after DMH1 treatment” Is this supposed to be a reference from previous work? This hasn’t been shown in this manuscript yet.

Sorry for the confusion, but we sometimes summarized the results data briefly before going through each piece of data to support the statement, in hopes of more clearly communicating to the reader the main conclusions from each results section.

That the drug and DNBMP treatments rescue fam mutants is convincing. “The amount of perichondral bone, such as seen in either the ceratohyal or hyomandibular, appeared reduced in the craniofacial skeleton of DMH1-treated larvae at 7dpf, compared to DMSO-treated 203 controls (Fig. 5J-K’’)” I would point out the opercle and branchiostegal ray dermal bones are unaffected. This is an excellent internal

© 2024. Published by The Company of Biologists under the terms of the Creative Commons Attribution License (https://creativecommons.org/licenses/by/4.0/).

control demonstrating tissue specific defects and not some global change. On this note, can you perform some quantification of dermal bone mineralization score that would be unchanged to drive this point home?

Thanks for raising this interesting point. We are actually preparing a manuscript to focus more on dermal bone development in these Bmp inhibition models, so we prefer to respond to this insightful comment by simply mentioning the observation that those dermal bones (opercle and branchiostegal rays) appear to be unaffected, reflecting specificity of this particular DMH1 treatment protocol on endochondral ossification (lines 255-258). Since the reviewer demonstrates interest in this subject, we actually think that we are seeing an effect of Bmp inhibition on a few bones that are classified as "dermal bones", but they initiate in the perichondrium, and flare out into the dermis, such as the dentary and quadrate.

The heat shock induction experiment in supplemental fig. 3 is good, but an important control is missing, the non heat shock transgene to show the amount of leakiness from this transgene without heat shock. Especially because this condition is presented in figure 6 it is important to know if there are basal levels of dnBMPR in these conditions, I suspect there is since heat shock driven transgenes can sometimes be expressed in the absence of heat shock. In fact, in fig 6B there may be reduced p-Smad staining reflecting a constitutive, low level, blockade in this genotype. Because timing is so important to this model, if the treatment really is acute or sustained is an important point.

Thanks for pointing out our omission of an important control in this experiment. In response, we revised Supplemental Fig 3, adding this control, which actually did not appear to demonstrate any basal GFP expression.

All zebrafish images should be oriented per convention with anterior to the left. This makes the images and the figures easier for the reader to interpret.

Corrected.

A couple of sentences early in the introduction describing exactly what proteoglycans are would be helpful for non-expert, general, readers of Development. Core proteins, repeating glycosaminoglycans etc.

Yes, thanks for this nice suggestion, which was implemented (lines 94-96), also referring to new Figure 1A, which illustrated PG structure.

Second decision letter

MS ID#: DEVELOP/2023/201716

MS TITLE: Proteoglycan inhibition of canonical BMP-dependent cartilage maturation delays endochondral ossification

AUTHORS: Elham Koosha, Connor T. A. Brenna, Amir M. Ashique, Niteesh Jain, Katie Ovens, Toshiyasu Koike, Hiroshi Kitagawa and B. Frank Eames

I have now received all the referees reports on the above manuscript, and have reached a decision. The referees' comments are appended below, or you can access them online: please go to BenchPress and click on the 'Manuscripts with Decisions' queue in the Author Area.

The overall evaluation is positive and we would like to publish a revised manuscript in Development, provided that the referees' comments can be satisfactorily addressed. Please attend to all of the reviewers' comments (please also see Editor's Note, appended below) in your revised manuscript and detail them in your point-by-point response. If you do not agree with any of their criticisms or suggestions explain clearly why this is so. If it would be helpful, you are welcome to

© 2024. Published by The Company of Biologists under the terms of the Creative Commons Attribution License (https://creativecommons.org/licenses/by/4.0/).

contact us to discuss your revision in greater detail. Please send us a point-by-point response indicating your plans for addressing the referees' comments, and we will look over this and provide further guidance.

Reviewer 1

Advance summary and potential significance to field

See previous review

Comments for the author

The authors have largely addressed my previous concerns. However, I still have concern with the final sentence of the abstract: "These results suggest that osteoarthritis, which involves loss of cartilage PGs, ectopic cartilage maturation, and adjacent ectopic bone, might be due to aberrant PG-dependent growth factor signalling." While I agree that there may be similarities between endochondral ossification and joint cartilage biology, the current study in no way directly addresses the roles of proteoglycans or Bmp signaling in joint cartilage or osteoarthritis. This final sentence is therefore misleading. It is fine to discuss parallels to and implications for osteoarthritis in final paragraph of Discussion, but I think reference to osteoarthritis should be deleted from the abstract so as to not give the impression that this study addressed joint biology.

Reviewer 2

Advance summary and potential significance to field

Same as my first summary.

Comments for the author

I'm satisfied with the revised manuscript.

Reviewer 3

Advance summary and potential significance to field

The authors have done a nice job addressing my previous concerns. The new data controls, and language changes improved this manuscript which will be interesting for the Development readership. I offer only two minor suggestions below that will further strengthen this work.

Comments for the author

The manuscript is suitable for publication in its current form, but the following minor suggestions will further improve the work.

1. The new laser capture RNA seq is a nice addition and confirms that all the required components of the BMP pathway are present in chondrocytes. However, I do not see anywhere where the authors explicitly address my previous concern regarding which cells are the BMP signal sending vs signal receiving in this system. Since the authors discovered that the ligands/receptors and mediators are all in the same population do they interpret that this is autocrine BMP signaling? That is, is the production and secretion of BMP by chondrocytes followed by binding of BMP to BMP receptors on the same (secreting) cells? I realize additional experiments are required to rigorously test this model, but it seems that authors have sufficient data to at least interpret an autocrine (or paracrine?) mechanism in this system. Adding this interpretation to their model brings increased richness to the study.

© 2024. Published by The Company of Biologists under the terms of the Creative Commons Attribution License (https://creativecommons.org/licenses/by/4.0/).

2. The labels in fig 3 A,B,E, and F prevent the reader from seeing the ceratohyal in the low magnification view. Please move the labels use a line or some other way to indicate the element so the reader can actually see the data.

Second revision

Author response to reviewers' comments

2nd Response to Reviewers

Koosha et al. DEVELOP/2023/201716

Editor's Note:

Reviewer previously suggested consolidation of figures in this manuscript, which has not yet been addressed. Please consider if some figures can be combined or presented as supplementary information.

We're sorry for omitting a response to this specific point. Many of our figures are already very large as is, and the overall number of figures is within normal range for *Development*, so we prefer to leave their organization as is, subject to approval from the copy editors prior to publication.

Reviewer 1 Advance Summary and Potential Significance to Field:
See previous review

Reviewer 1 Comments for the Author:
The authors have largely addressed my previous concerns. However, I still have concern with the final sentence of the abstract: "These results suggest that osteoarthritis, which involves loss of cartilage PGs, ectopic cartilage maturation, and adjacent ectopic bone, might be due to aberrant PG-dependent growth factor signalling." While I agree that there may be similarities between endochondral ossification and joint cartilage biology, the current study in no way directly addresses the roles of proteoglycans or Bmp signaling in joint cartilage or osteoarthritis. This final sentence is therefore misleading. It is fine to discuss parallels to and implications for osteoarthritis in final paragraph of Discussion, but I think reference to osteoarthritis should be deleted from the abstract so as to not give the impression that this study addressed joint biology.

OK, we disagree with this reviewer that the prior wording suggested that the study addressed joint biology directly, but we changed the last portion to "Therefore, PG inhibition of canonical BMP-dependent cartilage maturation delays endochondral ossification, and these results offer hope for the development of growth factor therapies for skeletal defects of PG diseases.".

***** Reviewer 2 Advance Summary and Potential Significance to Field:
Same as my first summary.

Reviewer 2 Comments for the Author:
I'm satisfied with the revised manuscript.

Thank you.

***** Reviewer 3 Advance Summary and Potential Significance to Field:
The authors have done a nice job addressing my previous concerns. The new data, controls, and language changes improved this manuscript which will be interesting for the Development readership. I offer only two minor suggestions below that will further strengthen this work.

© 2024. Published by The Company of Biologists under the terms of the Creative Commons Attribution License (https://creativecommons.org/licenses/by/4.0/).

Thank you.

Reviewer 3 Comments for the Author:
The manuscript is suitable for publication in its current form, but the following minor suggestions will further improve the work.

1. The new laser capture RNA seq is a nice addition and confirms that all the required components of the BMP pathway are present in chondrocytes. However, I do not see anywhere where the authors explicitly address my previous concern regarding which cells are the BMP signal sending vs signal receiving in this system. Since the authors discovered that the ligands/receptors and mediators are all in the same population do they interpret that this is autocrine BMP signaling? That is, is the production and secretion of BMP by chondrocytes followed by binding of BMP to BMP receptors on the same (secreting) cells? I realize additional experiments are required to rigorously test this model, but it seems that authors have sufficient data to at least interpret an autocrine (or paracrine?) mechanism in this system. Adding this interpretation to their model brings increased richness to the study.

Good suggestion; we have added the following lines to the Results in response:

“These data support the idea that BMP signalling can be autocrine within cartilage, since both BMP ligands and receptors are expressed by chondrocytes.” (lines 182-184) We also added the phrase “avoiding perichondral cells” to lines 176-177, to support the specificity of the expression data.

2. The labels in fig 3 A,B,E, and F prevent the reader from seeing the ceratohyal in the low magnification view. Please move the labels use a line or some other way to indicate the element so the reader can actually see the data.

Done

Third decision letter

MS ID#: DEVELOP/2023/201716

MS TITLE: Proteoglycan inhibition of canonical BMP-dependent cartilage maturation delays endochondral ossification

AUTHORS: Elham Koosha, Connor T. A. Brenna, Amir M. Ashique, Niteesh Jain, Katie Ovens, Toshiyasu Koike, Hiroshi Kitagawa and B. Frank Eames
ARTICLE TYPE: Research Article

I am satisfied with the response to review and the revision of the manuscript. The manuscript has been accepted for publication in Development, pending our standard ethics checks.

© 2024. Published by The Company of Biologists under the terms of the Creative Commons Attribution License (https://creativecommons.org/licenses/by/4.0/).
